# C-terminal extension of HSPB6 in a family with myopathy and cataract

**DOI:** 10.1093/hmg/ddaf175

**Published:** 2025-11-26

**Authors:** Jaakko Sarparanta, Per Harald Jonson, Anna Vihola, Helena Luque, Rocío-Nur Villar-Quiles, Tanya Stojkovic, Veronica Sian, Charlotte Walder, Tiina Suominen, Peter Hackman, Norma B Romero, Bruno Eymard, Bjarne Udd

**Affiliations:** Folkhälsan Research Center, Helsinki, Finland and Medicum, University of Helsinki, Biomedicum Helsinki, Haartmaninkatu 8, FI-00290 Helsinki, Finland; Folkhälsan Research Center, Helsinki, Finland and Medicum, University of Helsinki, Biomedicum Helsinki, Haartmaninkatu 8, FI-00290 Helsinki, Finland; Folkhälsan Research Center, Helsinki, Finland and Medicum, University of Helsinki, Biomedicum Helsinki, Haartmaninkatu 8, FI-00290 Helsinki, Finland; Folkhälsan Research Center, Helsinki, Finland and Medicum, University of Helsinki, Biomedicum Helsinki, Haartmaninkatu 8, FI-00290 Helsinki, Finland; Centre de Référence des Maladies Neuromusculaires, Institut de Myologie, Centre de Recherche en Myologie, Sorbonne Université, APHP, Hôpital Pitié-Salpêtrière, 47-83 boulevard de l'Hôpital, F-75651 Paris Cedex 13, France; Centre de Recherche en Myologie, GH Pitié-Salpêtrière, Sorbonne Université-Inserm UMRS974, 47-83 boulevard de l'Hôpital, F-75651 Paris Cedex 13, France; Centre de Référence des Maladies Neuromusculaires, Institut de Myologie, Centre de Recherche en Myologie, Sorbonne Université, APHP, Hôpital Pitié-Salpêtrière, 47-83 boulevard de l'Hôpital, F-75651 Paris Cedex 13, France; Centre de Recherche en Myologie, GH Pitié-Salpêtrière, Sorbonne Université-Inserm UMRS974, 47-83 boulevard de l'Hôpital, F-75651 Paris Cedex 13, France; Folkhälsan Research Center, Helsinki, Finland and Medicum, University of Helsinki, Biomedicum Helsinki, Haartmaninkatu 8, FI-00290 Helsinki, Finland; Folkhälsan Research Center, Helsinki, Finland and Medicum, University of Helsinki, Biomedicum Helsinki, Haartmaninkatu 8, FI-00290 Helsinki, Finland; Neuromuscular Research Center, Tampere University Hospital and Fimlab Laboratories, Teiskontie 35, FI-33520 Tampere, Finland; Folkhälsan Research Center, Helsinki, Finland and Medicum, University of Helsinki, Biomedicum Helsinki, Haartmaninkatu 8, FI-00290 Helsinki, Finland; Centre de Recherche en Myologie, GH Pitié-Salpêtrière, Sorbonne Université-Inserm UMRS974, 47-83 boulevard de l'Hôpital, F-75651 Paris Cedex 13, France; Institut de Myologie, Neuromuscular Morphology Unit, GH Pitié-Salpêtrière, 47-83 boulevard de l'Hôpital, F-75651 Paris Cedex 13, France; Centre de Référence des Maladies Neuromusculaires, Institut de Myologie, Centre de Recherche en Myologie, Sorbonne Université, APHP, Hôpital Pitié-Salpêtrière, 47-83 boulevard de l'Hôpital, F-75651 Paris Cedex 13, France; Centre de Recherche en Myologie, GH Pitié-Salpêtrière, Sorbonne Université-Inserm UMRS974, 47-83 boulevard de l'Hôpital, F-75651 Paris Cedex 13, France; Folkhälsan Research Center, Helsinki, Finland and Medicum, University of Helsinki, Biomedicum Helsinki, Haartmaninkatu 8, FI-00290 Helsinki, Finland; Neuromuscular Research Center, Tampere University Hospital and Fimlab Laboratories, Teiskontie 35, FI-33520 Tampere, Finland

**Keywords:** myopathy, cataract, heat shock protein, chaperone, phase separation

## Abstract

The small heat shock protein HSPB6 (a.k.a. Hsp20) is highly expressed in striated and smooth muscles. It modulates the oligomerization of its paralogs HSPB1 and CRYAB (HSPB5) and is involved e.g. in cytoskeletal regulation and autophagy. While HSPB6 variants have been implicated in cardiomyopathy, they have not been previously linked to neuromuscular disease. We report here a patient with late-onset myopathy and cataract, carrying *in cis* the novel *HSPB6* variant c.464delC and the common polymorphism c.488G > C, together resulting in the extended protein p.Pro155Argfs^*^25;p.Gly163Arg. The family history was consistent with dominant inheritance. The mutant protein showed decreased solubility due to phase separation propensity, and caused mislocalization of CRYAB and BAG3, and a decrease of HSPB1 in transfected cells. The patient’s muscle biopsy showed rimmed vacuoles and, in line with the functional studies, accumulation of HSPB6 and its interaction partners. The identified *HSPB6* variants are most likely the cause of the muscle disease in this family, thus identifying *HSPB6* mutations as a novel cause of vacuolar myopathy. Other reported HSPB6 variants causing a late frameshift or extension may cause disease in a similar fashion.

## Introduction

Small heat shock proteins (sHSPs) are a family of molecular chaperones represented in the human genome by ten members (HSPB1–10). While the canonical function of sHPSs is to act as first responders against protein aggregation, the family members also have different specialized functions in regulation of various proteins, as well as structural roles. The defining feature of sHSPs is the α crystallin domain (ACD) of ~ 80 amino acids. This is flanked by largely disordered N- and C-terminal extensions of variable lengths that account for the functional differences between the family members. sHSPs form homo- and heterodimers through the ACD, and canonical members further assemble to large, dynamic oligomers. Oligomerization depends on binding of the ACD to C-terminal anchoring motifs (I/V/L-X-I/V/L) that are found on many sHSPs, as well as interactions between the extensions [1, 2].

HSPB6 (also known as Hsp20) is a ubiquitously expressed sHSP that is most abundant in striated and smooth muscles, especially slow-twitch skeletal muscle where it may account for > 1% of total protein [3–5]. HSPB6 homodimerizes but does not readily form large oligomers on its own [6–8]. In contrast, it forms heterodimers and -oligomers with two other small heat shock proteins—HSPB1 and αB-crystallin (CRYAB, HSPB5)—modulating their oligomer size and chaperone activity [6–13]. While HSPB6 has been described as a poor chaperone [6], it shows variable client-dependent chaperone-like activity *in vitro* [6, 11, 14–17], and suppresses the aggregation of short polyglutamine proteins in transfected cells [18, 19]. The chaperone activity of HSPB6 towards α-synuclein was recently shown to be dependent on lipid binding [17]; whether this is a more general feature of HSPB6, remains to be clarified.

Among the sHSPs, HSPB6 is the only one known to be phosphorylated by cyclic-nucleotide-dependent kinases PKA and PKG, whose target site is Ser16 in the N-terminal domain [20–23]. Phosphorylation decreases chaperone activity of HSPB6 towards some clients [11, 24], but increases it towards α-synuclein [17], and promotes the interaction of HSPB6 with the 14-3-3 signalling regulators [24–27]. While *in vitro* studies using non-phosphorylatable (Ser16Ala) and phosphomimicking (Ser16Asp) HSPB6 mutants have indicated that phosphorylation is essential for the cardioprotective effects of HSPB6 (discussed below) [28, 29], constitutive cardiac expression of either Ser16Ala or Ser16Asp mutants is detrimental in mouse models, highlighting the importance of correct regulation [29, 30]. HSPB6 can also be acetylated at the C-terminal lysine residue, and this may promote phosphorylation of Ser16 or otherwise have overlapping functional consequences with the phosphorylation [31, 32].

HSPB6 has been reported to be involved in a variety of cellular functions (for a review, see [33–35]). Well established is its role in promoting smooth muscle relaxation through depolymerization of the actin cytoskeleton [20, 22, 25, 36], which has been suggested to depend on the regulation of cofilin phosphorylation status by 14-3-3 [37]. By regulating the 14-3-3 proteins, HSPB6 may have widespread effects also on other 14-3-3 partners [24–27]. In line with its high expression in the heart, HSPB6 has been widely studied for its cardioprotective functions [28, 38–41]. The protective effects in various cardiac stress situations *in vivo* and *in vitro* involve several molecular pathways. HSPB6 exerts antiapoptotic effects that have been attributed to suppressing Bax [38], ASK1-JNK/p38 [42], and NF-κB pathways [40], and boosting AKT signalling [39, 43].

The cardioprotective effects of HSPB6 can also partly be explained by stimulation of autophagy [41]. Through its interaction with the proautophagic protein beclin 1 (BECN1), HSPB6 both reduces proteasomal degradation of BECN1 and modulates its interaction with BCL2, thereby promoting autophagic activity through a dual mechanism [41]. In addition, HSPB6 can interact with the BAG3 (Bcl-2-associated athanogene 3), a co-chaperone that mediates selective autophagy and has a multitude of other functions in maintaining proteostasis [18, 44, 45]. As the preferential sHSP partner of BAG3 in physiological chaperone concentrations is HSPB8, the relevance of the HSPB6–BAG3 interaction in autophagy or other functions of BAG3 remains unclear [18]. However, in an experimental setting, the chaperone activity of HSPB6 towards Htt-43Q was found to be BAG3-dependent [18], and a functional connection between the proteins is also supported by reduced level of HSPB6 in BAG3 knockout and mutant models [46].

Despite the multiple described functions, global HSPB6 knockout mouse models are viable and apparently largely healthy [47, 48], suggesting functional redundancy with other sHSPs. However, HSPB6-deficient mice have been reported to have increased adiposity and energy expenditure due to boosted PPARγ signalling [47], and to show increased susceptibility to experimentally induced aortic dissection and rupture that may result from stiffening of actin cytoskeleton or loss of antiapoptotic functions in vascular smooth muscle cells [48]. Overt cardiac or skeletal muscle phenotypes have not been reported in the knockout models [47, 48].

Two HSPB6 missense variants, p.Ser10Phe and p.Pro20Leu, have been reported in the heterozygous state in patients with dilated cardiomyopathy, the latter being also found in the general population [41, 49]. Both of these variants reduce the thermal stability of HSPB6 and increase its chaperone activity *in vitro* [50], and have been found to abrogate the cardioprotective effects of HSPB6 in cell and animal models [41, 49]. The p.Ser20Leu variant was reported to interfere with phosphorylation of Ser16 [49] but the finding may reflect altered antibody binding to the mutant sequence as the variant protein was more rapidly phosphorylated by PKA *in vitro* [50]. The p.Ser10Phe variant, on the other hand, impairs the interaction of HSPB6 with BECN1, interfering with its ability to stimulate autophagy [41].

While variants in several other sHSPs (HSPB1, HSPB3, CRYAB, HSPB8) cause myo- and neuropathies (reviewed in [35, 51]), HSPB6 variants have thus far not been implicated in neuromuscular disease. We report here variants causing a C-terminal extension of the HSPB6 protein as a likely cause of dominant myopathy and cataract.

## Results

### Clinical features and ancillary tests

**Figure 1:**
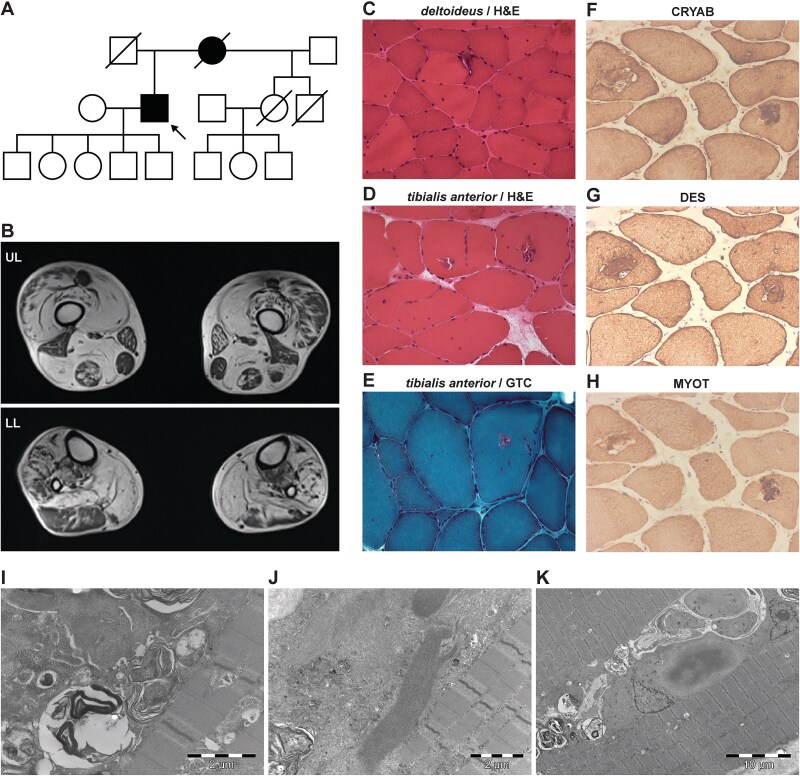
The family and clinical findings. A) The pedigree of the family, consistent with dominant inheritance. The index case is indicated by an arrow. B) Magnetic resonance imaging of the proband’s legs at age 57. The upper legs (UL) showed an involuted *quadriceps*, and sparing of *rectus femoris*, adductors, *semimembranosus*, *gracilis* and *sartorius*. The lower legs (LL) showed involvement of both anterior and posterior compartments, with sparing of *tibialis posterior*. C–E) Haematoxylin and eosin (H&E) and Gömöri trichrome (GTC) stainings from the proband’s *deltoideus* and *tibialis anterior* muscle biopsies showed unequal muscle fibre size, internalised nuclei, and several muscle fibres with rimmed vacuoles. F–H) On serial sections of *tibialis anterior* biopsy, immunocytochemical analysis for αB-crystallin (F), desmin (G) and myotilin (H) showed labelling of protein deposits around rimmed vacuoles. (I–K) Electron microscopy analysis demonstrated many muscle fibres containing abundant autophagic elements (I, K), dense filamentary inclusions (J, K) and tubulofilamentary inclusions characteristic of bordered vacuoles (J).

The index case is a male from the French Antilles (Fig. 1A). Motor development and motor performance in childhood and adolescence was normal. He developed cataract in the left eye at age 30. He reported proximal lower limb weakness since the age of 43 years, with difficulty in running and climbing stairs. At the first consultation at 48 years, he performed the 10-metre walking test unaided in 7 s.

Progressive lower limb weakness led to a limited walking perimeter, and since age 50 he started using walking aids (cane and shortly after a pair of crutches). Subsequently he also developed prominent axial weakness, contrasting with a preserved strength of upper limbs. Physical examination at age 57 revealed lower limb weakness, with marked involvement of proximal (*glutei*, *psoas*) but also distal muscles (*tibialis anterior, fibularis longus* and *brevis*). There was quadriceps hypertrophy and severe axial involvement with neck extensors and abdominal muscle weakness. There was no significant upper limb weakness. The 10-metre walking test was performed in 19 s, using crutches. He needed hand aid for rising from a chair. There was no facial, ocular or bulbar involvement. Cardiac evaluations were normal. Pulmonary function tests showed a normal respiratory function FVC (83% at age 57). He lost the ability to walk at 62 years of age, requiring wheelchair for ambulation.

Serum CK was mildly elevated (290–700 U/l). Electromyography suggested a myopathic process in tibial muscle and proximal muscles of upper limbs. Motor unit potentials were polyphasic, of reduced amplitude and short duration.

Muscle MRI confirmed axial involvement with marked atrophy and fatty replacement of paravertebral lumbar muscles. Lower limb involvement was patchy, with prominent atrophy and fatty replacement of *glutei*, *psoas*, great adductor, *sartorius*, *semimembranosus*, *soleus*, *tibialis anterior* and *fibularis longus* and *brevis* (Fig. 1B).

Muscle biopsies from *deltoideus* (performed at 48 years of age) and *tibialis anterior* (age 49 years) showed fibre size variation, fibre atrophy and the presence of rimmed vacuoles (Fig. 1C–E). There were no necrotic or regenerating fibres, and dystrophin, sarcoglycan, α-dystroglycan and dysferlin showed a normal expression pattern (not shown). However, immunocytochemical analysis of αB-crystallin, desmin and myotilin showed labelling of protein deposits around rimmed vacuoles (Fig. 1F–H). Electron microscopy investigations revealed a large number of muscle fibres containing abundant autophagic elements, dense filamentary inclusions and tubulofilamentary inclusions characteristic of rimmed vacuoles (Fig. 1I–K). There were also some abnormal mitochondria containing crystalline inclusions.

### Variants identified in the proband cause HSPB6 frameshift and extension

The family history was compatible with dominant inheritance (Fig. 1A). The proband’s mother had been operated for a cataract as a young adult. She had suffered from lower limb weakness since the sixth decade, needing a wheelchair since the age of 70 years. He had two half-siblings, with no muscle involvement according to the patient, but unfortunately, all these relatives are deceased and hence not available for genetic analysis nor clinical examination. The proband’s children (currently aged 29–47 years) have not been examined.

**Figure 2:**
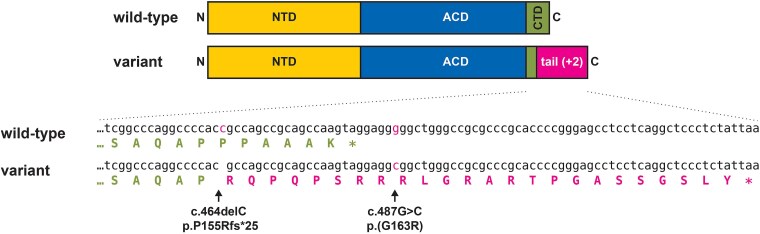
Identified HSPB6 variants. Top: The wild-type HSPB6 protein comprises the N-terminal domain (NTD), the α crystallin domain (ACD), and a short C-terminal domain (CTD) which is extended in the +2 frame in the p.P155Rfs^*^25;G163R variant protein. Bottom: cDNA and protein sequences of HSPB6, with the identified DNA variants and the expected consequences on the protein level indicated.

Pathogenic variants in the *VCP, RYR1* and *ZNF9* genes were excluded by Sanger sequencing. High-throughput sequencing (HTS) of a panel of 28 genes related to myofibrillar and vacuolar myopathies revealed no pathogenic variants in the index patient, but the larger MyoCap panel [52] identified 29 rare non-synonymous or possible splicing variants, 15 of which were classified as benign or likely benign on ClinVar. Of the remaining, the best candidate considering the dominant mode of inheritance, the muscle pathology, and the clinical phenotype was the previously unreported single-nucleotide deletion c.464delC in *HSPB6.* The variant causes a frameshift near the C-terminus of HSPB6, and extension of the protein (p.Pro155Argfs^*^25) (Fig. 2). In addition, a common polymorphism c.488G > C (rs11549030; MAF ≈ 0.15) was present in the 3’UTR of the *HSPB6* gene. According to HTS reads, the two variants were on the same allele, and this was confirmed by cloning and Sanger sequencing of PCR products (not shown). The normally silent c.488G > C variant causes a further Gly > Arg substitution in the C-terminal tail, the resulting protein being p.Pro155Argfs^*^25;p.Gly163Arg (Fig. 2).

**Figure 3:**
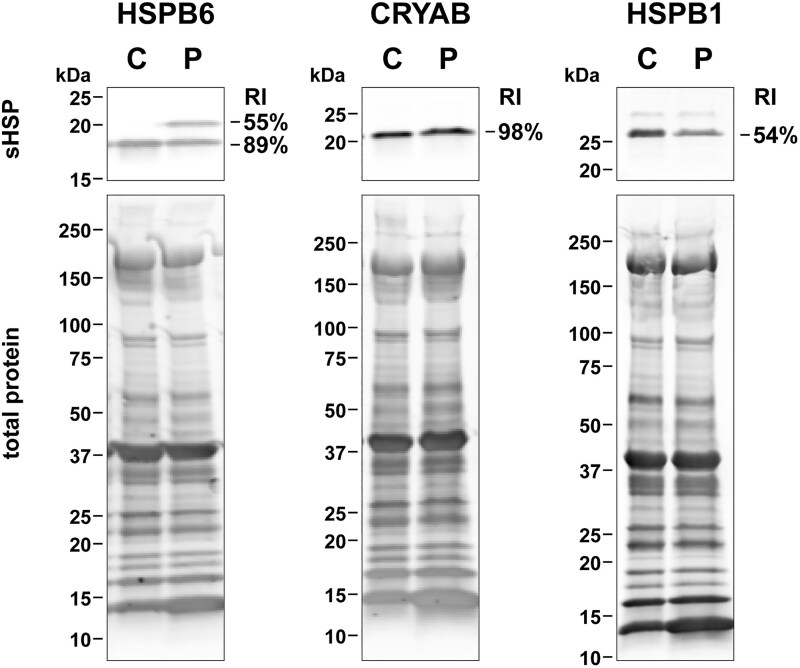
sHSP expression in proband’s muscle. The expression of HSPB6, CRYAB and HSPB1 in a pooled control sample (C) and the proband’s muscle extract (P) were analyzed by western blotting, with total protein staining as loading control. The relative intensities (RI) indicate the intensity of each sHSP band in the proband’s sample normalized to total protein and expressed as % of the pooled control. WB analysis confirmed the expression of the extended HSPB6 protein in the proband’s muscle. CRYAB expression in the proband was similar to control, whereas HSPB1 showed a ~ 50% reduction.

Western blotting (Fig. 3) showed the extended HSPB6 protein in the proband’s muscle biopsy sample at a level comparable to wild-type HSPB6, indicating that the mutant protein is expressed and stable. Of sHSPs regulated by HSPB6, the expression of CRYAB appeared normal, whereas the level of HSPB1 was reduced compared to a pooled control sample.

### Extended HSPB6 species show reduced solubility and form cytoplasmic foci

**Figure 4:**
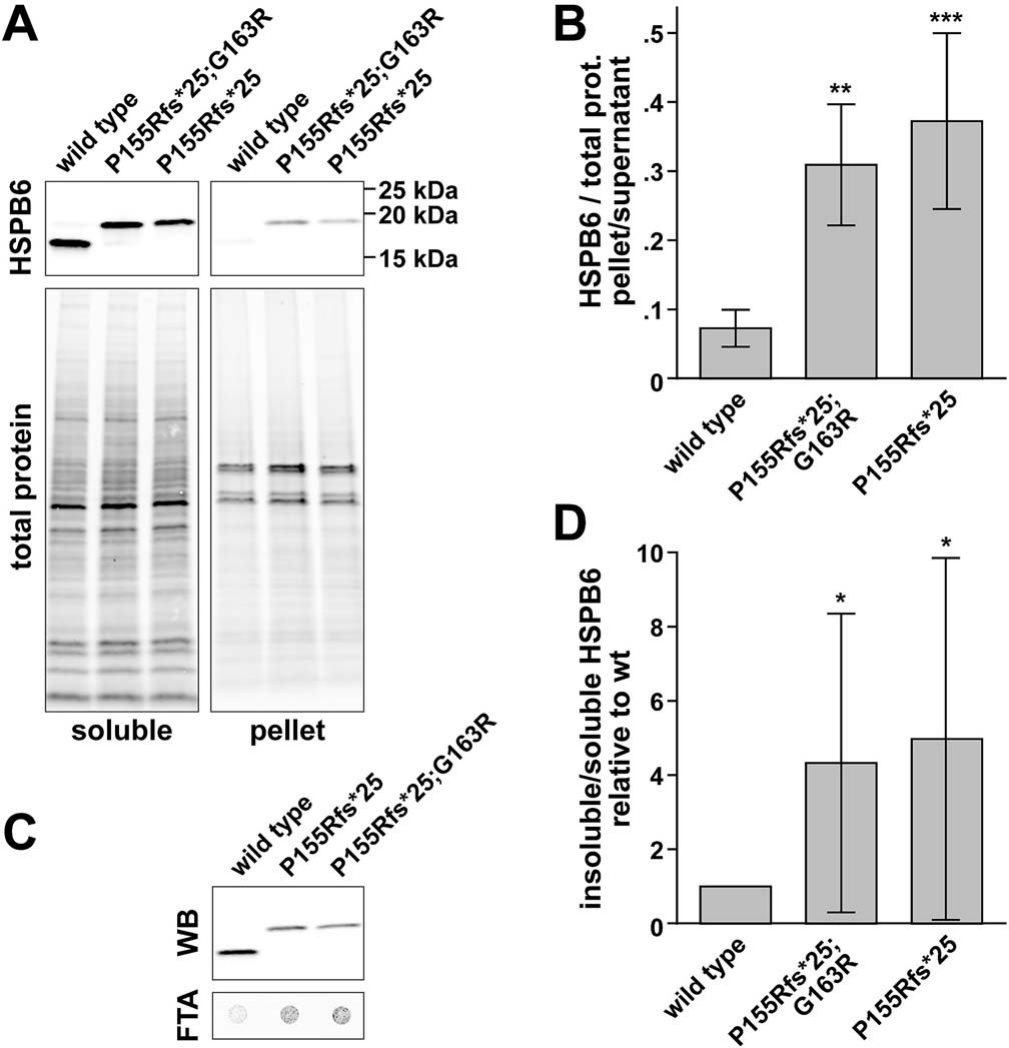
Solubility assays. HeLa cells expressing untagged wild-type or variant HSPB6 constructs were fractionated to RIPA-soluble insoluble fractions (A–B) or analysed by filter trap assay to detect SDS-insoluble aggregates (C–D). A) Representative western blotting of RIPA-soluble and insoluble (pellet) fractions with HSPB6 antibody and total protein staining. B) Quantification of the RIPA-insoluble/soluble HSPB6 ratio normalized to total protein (mean ± SD of *n* = 8 samples from two experiments). C) Representative western blotting (WB; soluble proteins) and filter trap membranes (FTA; SDS-insoluble proteins) stained for HSPB6. D) Quantification of the SDS-insoluble/soluble HSPB6 ratio, normalized to the mean of wild-type in each experiment (mean ± SD of *n* = 4 experiments, each consisting of 3–4 technical replicates). Asterisks (B, D) indicate significant differences compared to wild-type (^*^P < 0.05, ^**^P < 0.01, ^***^P < 0.001; Kruskal-Wallis test with Dunn’s multiple comparisons test).

To understand the effects of the identified variants on the HSPB6 protein, we expressed HSPB6 constructs in HeLa cells. Both p.Pro155Argfs^*^25 and p.Pro155Argfs^*^25;p.Gly163Arg constructs were included in the studies to dissect the relative contributions of the two variants to protein behaviour. Compared to wild-type HSPB6, both variant proteins showed decreased RIPA solubility in soluble/pellet fractionation and decreased SDS solubility in a filter trap assay (Fig. 4).

**Figure 5:**
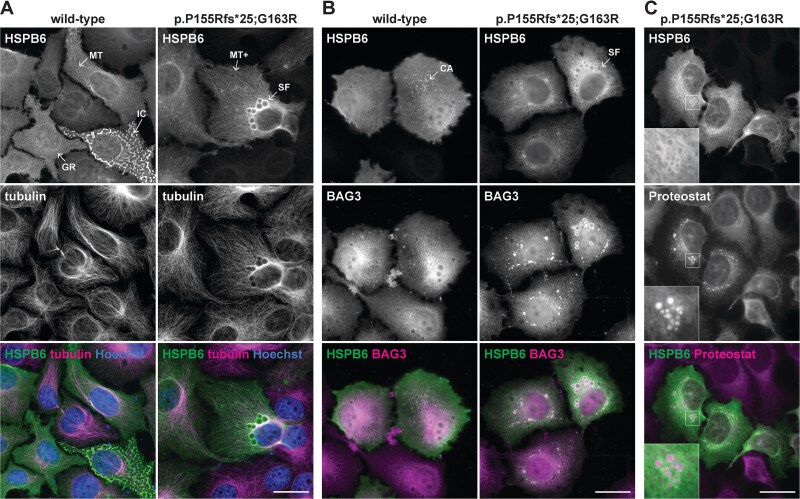
Localization of HSPB6 in HeLa cells. HeLa cells expressing untagged wild-type or p.P155Rfs^*^25;G163R HSPB6 constructs were stained for HSPB6 in combination with tubulin (A), BAG3 (B), the Proteostat dye (C), or HSPB1 (D–E). Wild-type HSPB6 showed diffuse localization with association to microtubules (MT) and cortical actin (CA), and in some cells (fixation-induced) separation to granules (GR) or irregular compartments (IC). The p.P155Rfs^*^25;G163R mutant constructs formed Proteostat-reactive spherical foci (SF) that recruited endogenous BAG3, and showed somewhat more pronounced microtubular association (MT+). The insets in C show a fourfold magnification of the boxed region. For the p.P155Rfs^*^25 construct and methanol-fixed samples, see Supplementary Fig. 1. Scale bars 20 μm.

In immunofluorescence (IF) microscopy of transfected HeLa cells, wild-type HSPB6 typically showed a diffuse cytoplasmic and nuclear localization, with some association to microtubules and cortical actin (Fig. 5, Supplementary Fig. 1A). In some cells with high expression, wild-type HSPB6 formed small cytoplasmic granules or separated as irregular compartments (Fig. 5); however, live-cell experiments (see below) suggested at least the former pattern to be fixation-induced. Both variant proteins showed, in addition to wild-type-like patterns, more prominent microtubular association and formed (near-)spherical structures ranging from punctate to several microns in diameter (Fig. 5, Supplementary Fig. 1B–C). These spherical foci—observed both with PFA and methanol fixation—appeared generally larger in p.Pro155Argfs^*^25-expressing cells, and smaller but more numerous with p.Pro155Argfs^*^25;p.Gly163Arg. The foci efficiently recruited endogenous BAG3 (Fig. 5B, Supplementary Fig. 1C). Especially larger foci showed a seemingly hollow core in immunostainings, likely due to inefficient antibody penetration. To study the internal composition of the foci, we employed Proteostat, a molecular rotor dye that binds the cross-beta structures found in protein aggregates and condensates [53, 54], and was previously shown to positively stain mutant HSPB8 aggregates [55]. Proteostat fluorescence observed throughout the HSPB6 foci (Fig. 5C, Supplementary Fig. 1C) indicated that these structures are comprised of protein with cross-beta conformation, most probably mutant HSPB6 itself.

### Variant HSPB6 undergoes phase separation

**Figure 6:**
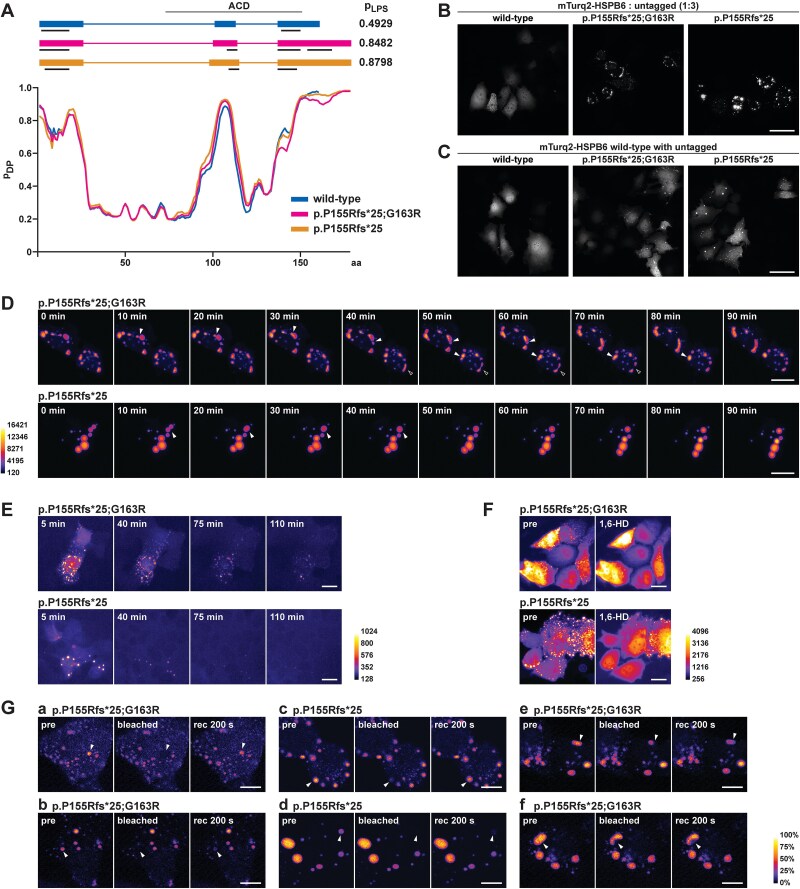
Phase separation. A) Prediction of HSPB6 phase separation propensity with the FuzDrop method. The traces show droplet-promoting probabilities (p_DP_) in the sequences of wild-type HSPB6 (blue), p.P155Rfs^*^25;G163R (magenta) and p.P155Rfs^*^25 (orange) as a function of amino acid number (aa). In the protein diagrams, shown in the same amino acid scale, thick lines indicate predicted droplet-promoting regions, with aggregation hot spots underlined in black. Both mutant proteins show an increased overall phase separation probability (p_LPS_) compared to wild-type. B) PFA-fixed HeLa cells transfected with the indicated mTurquoise2 (mTurq2)-tagged and untagged HSPB6 constructs (1:3 ratio). Scale bar 50 μm. For comparison with HSPB6 IF staining and mCherry-tagged constructs, see Supplementary Fig. 2. C) PFA-fixed HeLa cells transfected with wild-type mTurq2-HSPB6 and the indicated untagged constructs (1:3 ratio). The wild-type protein was recruited to the mutant HSPB6 foci. Scale bar 50 μm. D) Time-lapse microscopy of live HeLa cells transfected with mTurq2-tagged and untagged HSPB6 constructs at a 1:3 ratio. Fusion (closed arrowheads) and fission (open arrowheads) of foci demonstrate liquid behaviour of mutant HSPB6. Scale bars 20 μm. E) Time-lapse microscopy of HeLa cells after PEG-induced cell fusion shows dissolution of HSPB6 foci. Time stamps indicate time after PEG treatment. Scale bars 20 μm. F) The majority of mTurq2-HSPB6 foci dissolve upon 1,6-hexanediol (1,6-HD) treatment. Scale bars 20 μm. G) FRAP of mTurq2-HSPB6 foci. The frames show target cells before and immediately after photobleaching, and after a recovery of 200 s. some mutant HSPB6 foci showed near-complete fluorescence recovery during the follow-up period (a,c) whereas others hardly recovered (b,d). Inefficient recovery of some partially photobleached foci demonstrated limited HSPB6 mobility within foci (e,f). Intensity in each image series is scaled between the maximum and minimum fluorescence intensity within the series. Scale bars 10 μm. For FRAP recovery curves, see Supplementary Fig. 3.

The spherical appearance of mutant HPSB6 foci was suggestive of liquid–liquid phase separation. Indeed, analysis of wild-type and mutant HSPB6 protein sequences by the FuzDrop method [56] identified regions with a high droplet-promoting probability (p_DP_) (Fig. 6A). A droplet-promoting region predicted in the C-terminal part of HSPB6 was extended by the mutations, and FuzDrop predicted for the mutant proteins overall phase separation probabilities (p_LPS_) of > 0.8, which indicates that they are likely to drive liquid–liquid phase separation on their own [56]. Wild-type HSPB6, with p_LPS_ of 0.49 and several segments with p_DP_ > 0.6 is likely to act as a droplet client, i.e. undergo phase separation driven by additional interactions [56]. Furthermore, FuzDrop identified potential aggregation hot-spots which may promote conversion of phase-separated proteins into insoluble amyloids [57]. An additional such segment was predicted in HSPB6 p.Pro155Argfs^*^25;p.Gly163Arg compared to wild-type HSPB6 and p.Pro155Argfs^*^25 (Fig. 6A).

For studying the phase separation experimentally, we generated fluorescent HSPB6 constructs N-terminally tagged with mCherry or mTurquoise2 (mTurq2). To minimize the effects of the tags on protein behaviour, these were used in co-transfections with corresponding untagged constructs. Wild-type HSPB6 tagged with either fluorescent protein showed predominantly diffuse localization (Fig. 6B, Supplementary Fig. 2A). While a subset of PFA-fixed cells showed a granular pattern, this was never seen in live cells. Indeed, comparison of live and PFA-fixed cells demonstrated that the granules coalesce from diffuse HSPB6 during fixation (Supplementary Fig. 2B). In clear contrast to wild-type HSPB6, both the mCherry- and mTurq2-tagged mutant constructs localized to cytoplasmic foci. The mutant mTurq2-HSPB6 constructs showed pronounced localization to globular foci which were highly similar in shape and structure to those seen with the untagged constructs (Fig. 6B, Supplementary Fig. 2A). Notably, also wild-type mTurq2-HSPB6 partially localized to foci in cells co-transfected with untagged mutant constructs (Fig. 6C). mCherry-tagged HSPB6, while showing less pronounced propensity to form foci, altered the morphology of the foci (Supplementary Fig. 2A). We therefore chose to use mTurq2-tagged constructs in the subsequent studies.

Timelapse imaging of live cells showed that mutant mTurq2-HSPB6 foci formed and grew quickly with increasing cellular level of the proteins. Observed fusion and fission events demonstrated liquid droplet properties of the foci (Fig. 6D). Following PEG-induced cell fusion, leading to lowered local concentration of the protein, the foci could also be seen to dissolve, indicating that they are reversible (Fig. 6E). Similarly, the majority of the foci dissolved upon treatment with 1,6-hexanediol (Fig. 6F), as expected for phase-separated condensates [58]. In fluorescence recovery after photobleaching (FRAP) experiments (Fig. 6G, Supplementary Fig. 3), some of the mutant mTurq2-HSPB6 foci showed near-complete recovery during the follow-up period of 200 seconds, demonstrating that they can exchange material with their surroundings. Some foci, in contrast, showed little recovery. Furthermore, while some partially photobleached large foci recovered to nearly uniform intensity, others failed to do so. Altogether, our findings suggest that the mutant HSPB6 foci are initially liquid, but can over time become less dynamic, likely by acquiring aggregate- or gel-like properties.

### Variant HSPB6 decreases the HSPB1 level and alters sHSP localization

**Figure 7:**
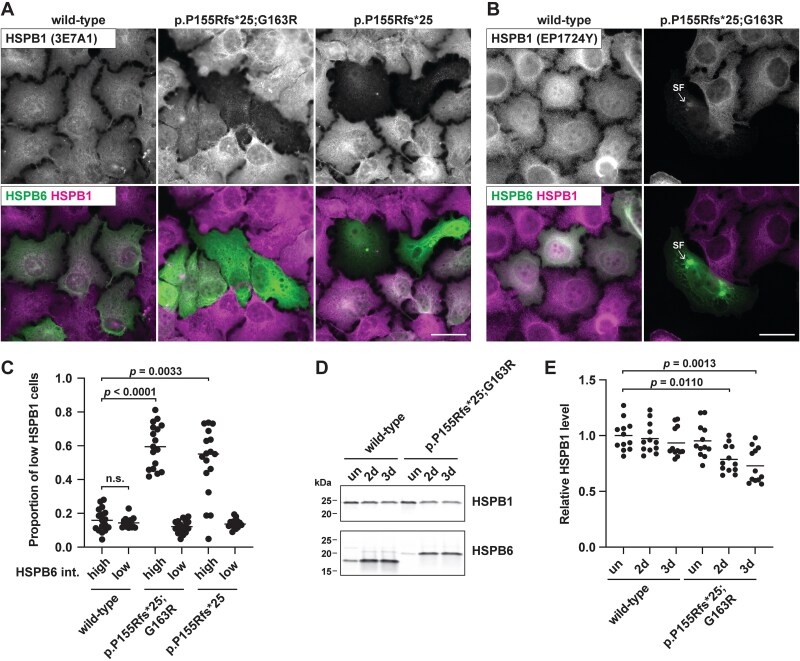
Decrease of HSPB1 by variant HSPB6. A–C) HeLa cells transfected with untagged wild-type or variant HSPB6 constructs were stained for HSPB6 with either of two HSPB1 antibodies. Cells expressing variant HSPB6 often showed a reduction in endogenous HSPB1, and association of remaining HSPB1 with the HSPB6 spherical foci (SF). C) High-content analysis of cells transfected and stained as in B. Cells with a high intensity of variant HSPB6 constructs showed an increased proportion of those with a reduced HSPB1 intensity compared to cells expressing wild-type HSPB6 and to low-HSPB6-intensity (i.e. untransfected) cells in the same wells. The datapoints represent *n* = 17 wells from three independent experiments, with medians indicated by horizontal lines. D–E) Stably transfected HeLa cells were induced to express wild-type or p.P155Rfs^*^25;G163R for 2 or 3 days or left uninduced (un). HSPB1 and HSPB6 expression were analyzed by western blotting. For total protein staining, see Supplementary Fig. 4A. E) Quantification of HSPB1 expression. Datapoints shows relative HSPB1 expression (normalized to total protein and to the mean of uninduced wild-type cells within the experiment); *n* = 12 wells from three independent experiments. For qPCR of *HSPB1* mRNA, see Supplementary Fig. 4B. The *p* values (C, E) indicate statistical significance according to Kruskal-Wallis test with Dunn’s multiple comparisons test.

Given the well-established hetero-oligomerization of HSPB6 with HSPB1 and CRYAB [6–13], we characterized the effects of variant HSPB6 on these sHSPs. Interestingly, HSPB1 showed a clear reduction in HeLa cells expressing extended HSPB6 constructs, with the remaining HSPB1 sometimes seen associated with the HSPB6 foci (Fig. 7A–C). A reduction in HSPB1 protein level was also seen in stably transfected HeLa cells induced to express HSPB6 p.Pro155Argfs^*^25;p.Gly163Arg (Fig. 7D–E, Supplementary Fig. 4A). This decrease was most likely caused by a protein-level effect, as wild-type and p.Pro155Argfs^*^25;p.Gly163Arg cells showed similar (albeit decreased) *HSPB1* mRNA expression (Supplementary Fig. 4B).

CRYAB could not be clearly detected by IF in HeLa cells using our antibody. To look at the effects of variant HSPB6 in a cell type expressing endogenous CRYAB, we used mRNA transfection to express wild-type and p.Pro155Argfs^*^25;p.Gly163Arg variant HSPB6 in L6 myotubes. While the microscopic analysis of these cells was complicated by the high levels of the endogenous proteins, often showing punctate patterns, infrequent myotubes expressing HSPB6 p.Pro155Argfs^*^25;p.Gly163Arg showed similar spherical foci as seen in HeLa cells, decorated by endogenous CRYAB (Supplementary Fig. 5).

**Figure 8:**
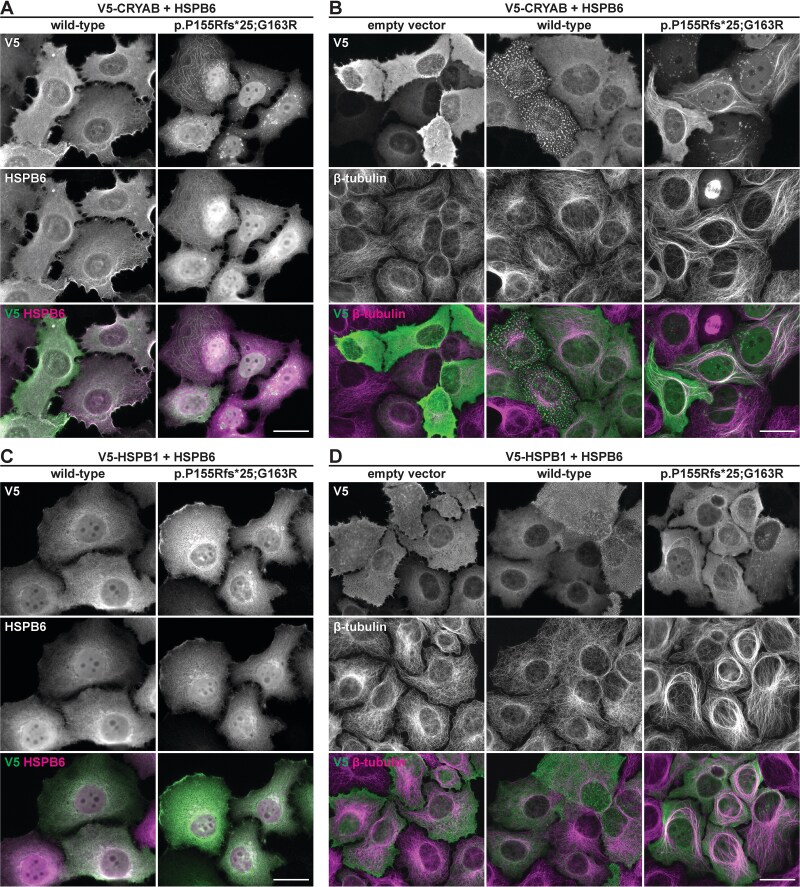
Effects of HSPB6 on cotransfected CRYAB and HSPB1. HeLa cells expressing V5-CRYAB (A–B) or V5-HSPB1 (C–D) in combination with wild-type or p.P155Rfs^*^25;G163R mutant HSPB6 or an empty vector were stained for the V5 tag and HSPB6 (A, C) or tubulin (B, D). Both sHSPs largely followed the localization of wild-type HSPB6. In cells expressing mutant HSPB6, V5-CRYAB localized to the HSPB6 foci and sometimes showed prominent targeting to microtubules accompanied by seemingly altered microtubular organization. V5-HSPB1 showed similar but on average less pronounced behavior. For the p.P155Rfs^*^25 construct and cotransfections with GFP-V5, see Supplementary Fig. 6. Scale bars 20 μm.

Possible dominant effects of HSPB6 variants on HSPB1 and CRYAB were further characterized in a forced co-expression setup in HeLa cells (Fig. 8, Supplementary Fig. 6). In single transfections and in co-transfections with wild-type HSPB6, V5-tagged CRYAB and HSPB1 showed diffuse localization and association with the actin cytoskeleton, largely co-localizing with wild-type HSPB6. In contrast, when co-transfected with variant HSPB6 constructs, V5-CRYAB localized prominently to the spherical HSPB6 foci and, in a small subset of cells, showed striking microtubular association (Fig. 8A–B, Supplementary Fig. 6). In these cells, microtubule organization often appeared altered, with thick non-centrosomal microtubule bundles. We found no obvious evidence for increased microtubule content in cells with microtubular V5-CRYAB association, nor for stabilization of microtubules under nocodazole or cold treatments (not shown).

Co-transfected V5-HSPB1 similarly showed association with mutant HSPB6 foci and microtubules, although this appeared somewhat less prominent than observed with V5-CRYAB. (Fig. 8C–D, Supplementary Fig. 6A–B). For comparison, microtubular alterations were not observed in cells co-expressing V5-tagged green fluorescent protein (GFP-V5) with HSPB6 variants, and GFP-V5 did not show pronounced association with HSPB6 foci (Supplementary Fig. 6C). Altogether, these findings demonstrate that variant HSPB6 can have pleiotropic effects on its partner sHSPs, with conceivable downstream consequences ranging from loss-of-function (via dysregulation, decreased protein level, or chaperone sequestration) to gain-of-function (via microtubular alterations).

### Proband’s myopathology is consistent with HSPB6 aggregation

**Figure 9:**
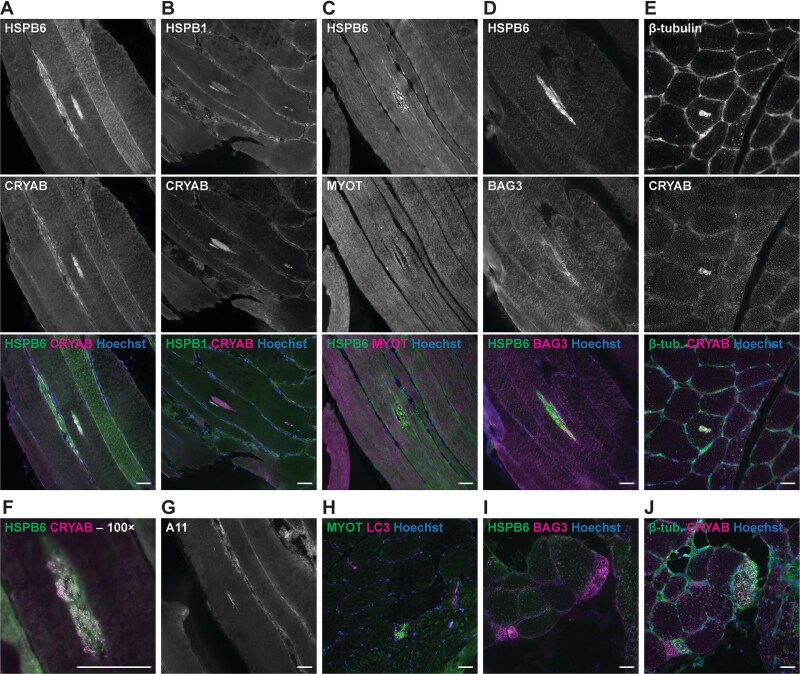
Immunofluorescence microscopy of the proband’s muscle. Immunofluorescence microscopy of the proband’s *tibialis anterior* biopsy revealed protein accumulations positive for HSBP6 (A, C, D, F, I), CRYAB (A, B, E, F), HSPB1 (B), β-tubulin (E), and the A11 oligomer antibody (G), and variably for BAG3 (D) but negative for myotilin (C). At high magnification, the accumulations had a granular appearance (F). Individual atrophic fibres showed increased reactivity for BAG3 (I) or β-tubulin (J), or myotilin-positive cytoplasmic bodies (H). Scale bars 50 μm.

To further characterize the myopathology and to correlate that with the findings from our functional studies, we analysed the proband’s *tibialis anterior* biopsy by immunofluorescence microscopy (Fig. 9). This revealed, consistently with the immunohistochemical analyses, central protein accumulations containing HSPB6, HSPB1, BAG3, and β-tubulin in addition to CRYAB, and positive for the A11 antibody which recognizes preamyloid oligomers. At high magnification, these accumulations showed a round-granular structure. While the sHSP-containing accumulations did not stain positive for myotilin in the analysed sections, some fibres contained separate myotilin-positive cytoplasmic bodies, In addition, individual fibres showed increased reactivity for BAG3 or β-tubulin.

### Reported HSPB6-extending variants have variable consequences and some may be pathogenic

**Figure 10:**
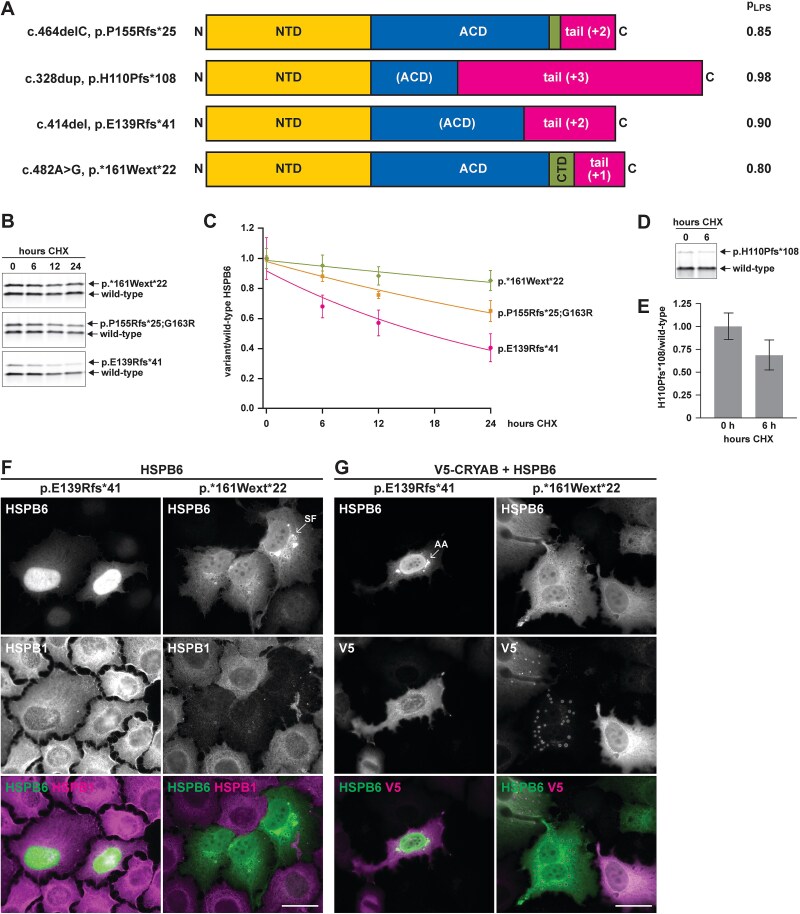
Analysis of reported HSPB6-extending variants. A) A schematic view on the variant HSPB6 proteins chosen for characterization, showing the reading frames of the extended tails, and the phase separation probabilities (p_LPS_) of the proteins calculated with the FuzDrop method. The p.P155Rfs^*^25 variant is shown for comparison. B–C) HeLa cells cotransfected with wild-type and variant HSPB6 (50:50 ratio) were treated with cycloheximide (CHX) for the indicated times. The graph (C) shows the remaining amount of variant HSPB6 normalized to the wild-type protein, with the ratio *t* = 0 set to 1 for each construct (mean ± SD of *n* = 6 replicates from two independent experiments). D–E) HeLa cells cotransfected with wild-type and p.H110Pfs^*^108 HSPB6 (20:80 ratio) were treated with cycloheximide (CHX) for 6 h. the graph (E) shows the remaining amount of variant HSPB6 normalized to the wild-type protein, with the ratio at *t* = 0 set to 1 (mean ± SD of *n* = 12 replicates from three independent experiments). For total protein stainings, see Supplementary Fig. 7. F–G) HeLa cells were transfected with HSPB6 constructs alone (F) or with V5-CRYAB (G) and stained for HSPB6, HSPB1 and V5 as indicated. In the few visibly transfected cells, HSPB6 p.E139Rfs^*^41 mostly showed diffuse, predominantly nuclear, localization, with amorphous aggregates (AA) observed very infrequently. HSPB6 p.^*^161Wext^*^22 formed spherical foci (SF) that recruited V5-CRYAB, and caused a reduction of endogenous HSPB1. Scale bars 20 μm.

While the HSPB6 frameshift identified in our proband is absent from the gnomAD database, a total of 28 other heterozygous frameshift or extending variants in HSPB6 were reported in gnomAD by January 2025. In 12 of these (Table 1), the new stop codon is late enough so as not to trigger nonsense-mediated decay (i.e. later than ~ 50 bases before the last exon junction), and they could hence give rise to abnormal proteins. To evaluate their potential pathogenicity, we studied in cell culture experiments the behaviour of three variant proteins with different characteristics (Fig. 10). Of the selected variants, c.328dup (p.His110Profs^*^108) causes an early frameshift and a very long tail of 107 amino acids in a frame different from our proband’s variant. The c.414del (p.Glu139Argfs^*^41) variant, present in several individuals in gnomAD, causes an out-of-frame tail in the same reading frame as ours, but somewhat longer, with the frameshift occurring late in the ACD. As a representative of the two stop-loss variants reported in gnomAD, both found in several individuals, we chose c.482A > G (p.^*^161Trpext^*^22). The FuzDrop method predicted increased phase separation probability for all three variant proteins (Fig. 10A).

**Table 1:** Reported HSPB6-extending variants.

**Transcript change**	**Protein change**	**Allele count**	**Allele frequency**	**Tail frame**
c.108dup	p.Leu37Alafs^*^73	1	6.50E-07	+3
c.141dup	p.Thr48Hisfs^*^62	2	1.30078E-06	+3
c.157dup	p.Tyr53Leufs^*^57	4	2.60197E-06	+3
c.266_267del	p.Ala89Glyfs^*^20	1	6,26E-07	+3
c.277del	p.Val93Trpfs^*^40	1	6.26E-07	+2
c.320del	p.Pro107Argfs^*^26	5	3.1631E-06	+2
c.328dup	p.His110Profs^*^108	2	1.30261E-06	+3
c.343del	p.Arg115Alafs^*^18	1	6.52E-07	+2
c.322-42_374dup	p.Val127Argfs^*^38	1	6.52E-07	+2
c.414del	p.Glu139Argfs^*^41	19	1.24016E-05	+2
c.479_480insTT	p.Lys160Asnfs^*^21	1	6.70E-07	+2
c.482A > C	p.^*^161Serext^*^22	17	1.42852E-05	+1
c.482A > G	p.^*^161Trpext^*^22	23	1.9334E-05	+1

gnomAD v4.1.0 Jan 28, 2025. The table lists variants affecting NM_144617.3 and not expected to cause nonsense-mediated mRNA decay.

When transfected to HeLa cells, the two variant proteins with frameshifts within the ACD showed lower expression levels than the other constructs. The level of p.Glu139Argfs^*^41 was typically 10–20% of the wild-type construct, while p.His110Profs^*^108 was hardly detectable in our regular setup (not shown). Cycloheximide chase assays confirmed quicker turnover of these variant proteins compared to wild-type HSPB6 (Fig. 10B–E, Supplementary Fig. 7).

The p.His110Profs^*^108 variant was omitted from microscopy studies due to protein instability. Of the remaining variant proteins, p.Glu139Argfs^*^41 was detectable only in a small fraction of cells; in these, it showed diffuse, mostly nuclear localization, with amorphous aggregates detected in extremely rare cells. It did not appear to affect endogenous HSPB1 nor cotransfected V5-CRYAB (Fig. 10F–G). The p.^*^161Trpext^*^22 protein, on the other hand, behaved very similarly to p.Pro155Argfs^*^25, forming prominent spherical foci that recruited V5-CRYAB, and causing a reduction in endogenous HSPB1 (Fig. 10F–G). However, in cotransfections with HSPB6 p.^*^161Trpext^*^22 and V5-CRYAB, we did not observe any cells with a microtubular phenotype.

## Discussion

Our index case was found to carry two *HSPB6* variants, c.464delC and c.488G > C, together resulting in a C-terminally extended mutant protein p.Pro155Argfs^*^25;p.Gly163Arg. Although in the absence of segregation data the causality cannot be proven with full certainty, the clear abnormal behaviour of the mutant protein in functional studies and the consistent molecular phenotype in the patient strongly suggest that the *HSPB6* variants underlie the myopathy in our family. The extended HSPB6 protein showed exaggerated phase separation propensity and affected the localization of its partners CRYAB, HSPB1 and BAG3 as well as wild-type HSPB6 in cultured cells, and this was reflected by accumulation of the same proteins in patient myofibres. Cells expressing extended HSPB6 also showed a reduction in HSPB1, again in line with the lower HSPB1 amount seen in patient muscle. The pathogenicity of the variant gains further support from the similar *in vitro* behaviour of the (neuro)myopathy-causing extension variants in HSPB8 [55].

Variant HSPB6 may cause muscle disease through one of several potential downstream mechanisms, or their combination. First, HSPB6 accumulation may interfere with protein quality control through sequestration of other chaperones and/or reduction of HSPB1 level. Second, the pathomechanism could involve a dominant toxic effect mediated by mutant HSPB6 itself or by abnormal behaviour of other sHSPs. Indeed, given the high expression of CRYAB and HSPB1 in muscle, any perturbations in their oligomer structure or dynamics are potentially harmful. Several disease-causing CRYAB and HSPB1 variants have been reported to cause altered oligomer size or stability (reviewed in [51]). Moreover, some variants in CRYAB affect its interaction with HSPB6 [59–61].

The microtubular phenotype observed in our transfection experiments is in line with the reported microtubule association of CRYAB and HSPB1, and with the effects of these sHSPs on microtubule structure and dynamics [62–65]. While the available biopsy material did not allow detailed analysis of microtubule structure in patient muscle, β-tubulin did localize to the sHSP accumulations within myofibres. Microtubular perturbations could hence be envisioned to play a role in the pathomechanism. Microtubules have been previously implicated in the pathogenesis of centronuclear myopathy and Duchenne muscular dystrophy [66–68]. Moreover, some CMT-causing HSPB1 variants cause increased binding to tubulin and microtubules, leading to microtubule stabilization that may cause disease by compromising axonal transport and autophagy in neurons [69, 70].

Both patients in our family were affected with cataract in addition to myopathy. Given that HSPB6 is expressed in the lens [3, 4], and that primary CRYAB variants are a known cause of hereditary cataract [51, 71–73], it is highly likely that the cataract in our patients is caused by the HSPB6 variant, possibly through a CRYAB-mediated mechanism. Moreover, since pathogenic variants in other sHSPs (HSPB1, HSPB3, CRYAB, HSPB8) can result in both myopathy and motor neuropathy phenotypes [35, 51] and HSPB6 shows some expression in neural tissues [3, 4, 74], HSPB6 variants could conceivably also be involved in peripheral neuropathy. Nevertheless, clinical examination and ENMG studies did not reveal any signs of peripheral nerve involvement in our index patient.

In conclusion, the identified *HSPB6* variants are the most likely cause of the late-onset vacuolar myopathy and cataract in our family. Since the double (p.Pro155Argfs^*^25;p.Gly163Arg) and single (p.Pro155Argfs^*^25) variant proteins showed largely comparable behaviour in our functional assays, we do not believe the c.488G > C variant to make a major difference in terms of pathogenicity.

Several other variants causing a frameshift and/or extension of HSPB6 have been reported. Our studies on representative variant proteins suggest that frameshifts disrupting the ACD lead to protein instability. Such loss-of-function alleles are unlikely to cause a dominant disease but could in homozygous individuals lead to a recessively inherited HSPB6 deficiency. The p.^*^161Trpext^*^22 variant, on the other hand, showed very similar behaviour to the extension identified in our family. Such stop-loss variants, as well as late frameshifts occurring after the ACD, could potentially be dominantly pathogenic.

## Materials and methods

### Clinical investigations

The index case was initially examined and followed-up by three of the authors on the Neuromuscular Reference Center in Pitié-Salpêtrière Hospital in Paris (France). Whole-body muscle MRI was performed for the index case and analysed by an expert radiologist. Two muscle biopsies were conducted and processed for standard histological and immunochemical studies, as previously described [75]. Informed consent was obtained, in agreement with local ethic committees and with the 1964 Helsinki declaration and its later amendments**.**

### Molecular genetics

Targeted high-throughput sequencing of the proband’s DNA was performed with a myofibrillar and vacuolar myopathy panel covering 28 genes, and subsequently with the MyoCap panel [52] version 4, covering 297 reported or potential myopathy genes (Supplementary table). To confirm the phase of the identified *HSPB6* variants, a PCR product spanning both variants in *HSPB6* exon 3 was amplified from the proband’s DNA, cloned with the Zero Blunt TOPO PCR cloning kit (Thermo Fisher Scientific) and Sanger sequenced. The *HSPB6* reference sequences used are RefSeq NM_144617.3 (mRNA) and RefSeq NP_653218.1 (protein).

### Antibodies

The following primary antibodies were used in the study: αB-crystallin mouse (ms) monoclonal antibody (mAb) G2JF (Leica Biosystems, NCL-ABCrys-512, RRID:AB_442024); αB-crystallin goat polyclonal antibody (pAb) (GeneTex, GTX88907, RRID:AB_10723662); BAG3 rabbit (rb) pAb (Proteintech, 10 599–1-AP, RRID:AB_2062602); β-tubulin rb pAb (Abcam, ab6046, RRID:AB_2210370); desmin ms mAb D33 (Dako/Agilent, M0760, RRID:AB_2335684); HSPB1 (Hsp27) ms mAb 3E7A1 (Proteintech, 66 767–1-Ig, RRID:AB_2882113); HSPB1 (Hsp27) rb mAb EP1724Y (Abcam/Epitomics, 2174–1, RRID:AB_991744); HSPB6 ms mAb 6A4 (Abnova, H00126393-M03, RRID:AB_10615179); HSPB6 rb mAb [EPR14458] (Abcam, ab184161, RRID:AB_2833086); HSPB6 rb pAb (Atlas Antibodies, HPA054811, RRID:AB_2682610); LC3 ms mAb 4E12 (MBL International, M152–3, RRID:AB_1279144); myotilin ms mAb RSO34 (Abnova, MAB9563, RRID:AB_10754717 or Leica Biosystems, NCL-Myotilin, RRID:AB_563903); myotilin rb pAb (Proteintech, 10 731–1-AP, RRID:AB_2297956); oligomer A11 rb pAb (Thermo Fisher, AHB0052, RRID:AB_2536236); V5 ms mAb (Thermo Fisher, R960–25, RRID:AB_2556564).

Secondary antibodies conjugated to Alexa Fluor (AF) 488, 546, 594, or 647 (Thermo Fisher) were used in immunofluorescence microscopy.

### Muscle histology and immunofluorescence

Haematoxylin & eosin and Gömöri trichrome stainings and immunohistochemical analyses for CRYAB, DES and MYOT were done using standard methodology. Electron microscopy analyses were performed as described [76]. Immunofluorescent stainings were done for HSPB6, CRYAB, HSPB1, myotilin, BAG3, LC3, and oligomer A11, and images were acquired with Zeiss Axio Imager M2 using a 20× NA 0.8 air objective and a 100× NA 1.3 oil immersion objective.

### SDS-PAGE and western blotting

Tibialis anterior muscle biopsy of the proband was homogenized in sample buffer (50 mM Tris–HCl pH 6.8; 4% SDS, 8.7% glycerol, 10% 2-mercaptoethanol, bromophenol blue) and heated at 98°C for 5 min. The sample was placed on ice and centrifuged, and the supernatant was stored in −80°C until used. A pooled control homogenate was similarly prepared from skeletal muscle biopsies from several individuals free of neuromuscular disease.

Protein samples were run in TGX minigels (Bio-Rad, Hercules, CA, USA) and transferred on nitrocellulose membranes with the Trans-Blot Turbo system (Bio-Rad). Total protein was stained with the Revert 700 or Revert 520 Total Protein Stain (Li-Cor Biosciences, Lincoln, NE, USA). Blots were stained with primary antibodies as specified, followed by fluorescently labelled secondary antibodies. Images were acquired with Odyssey Classic or Odyssey M scanners (Li-Cor) and quantified with Empiria Studio (Li-Cor) or Fiji (ImageJ2 2.14.0) [77].

### Plasmid constructs

Untagged and N-terminally V5-tagged HSPB6, CRYAB, and HSPB1 constructs in pCDNA5/FRT/TO [19] were a kind gift from Harm Kampinga. To introduce the part of *HSPB6* 3’UTR coding for the C-terminal extension, an oligonucleotide duplex was inserted to the wild-type HSBP6 constructs. The c.464delC; p.Pro155Argfs^*^25 and c.488G > C; p.Gly163Arg variants were then introduced by site-directed mutagenesis. To create mTurquoise2-HSPB6 and mCherry-HSPB6 constructs, the coding sequences of the fluorescent proteins were inserted to the respective untagged HSPB6 constructs with the NEBuilder HiFi assembly kit (New England Biolabs). For pCR-Blunt II-TOPO constructs, the inserts for wild-type and p.Pro155Argfs^*^25;p.Gly163Arg HSPB6 were PCR-amplified and cloned with the Zero Blunt TOPO PCR Cloning Kit (Thermo Fisher) in the orientation placing the T7 promoter upstream of the insert. For PiggyBac constructs, the inserts for wild-type and p.Pro155Argfs^*^25;p.Gly163Arg HSPB6 were inserted by restriction/ligation cloning between the EcoRI and NotI sites of the PB-CuO-CMV-MCS-EF1a-CymR-T2A-Puro vector (System Biosciences PBQM800A-1).

Constructs for the HSPB6 variants p.His110Profs^*^108, p.Glu139Argfs^*^41, and p.^*^161Trpext^*^22 were assembled on the PmlI/PspOMI-linearized pCDNA5/FRT/TO-HSPB6 backbone using the NEBuilder HiFi assembly kit, and insert fragments amplified from pCDNA5/FRT/TO-HSPB6 or human genomic DNA.

### Phase separation prediction

Phase separation and aggregation propensity of wild-type and variant HSPB6 was predicted with the FuzDrop method (https://fuzdrop.bio.unipd.it/predictor) [56].

### Protein solubility assays

HeLa cells were transiently transfected with untagged wild-type or mutant HSPB6 constructs for two days. For RIPA solubility assays, the cells were pelleted in PBS, lysed in RIPA (50 mM Tris–HCl pH 8.0, 150 mM NaCl, 1% Triton X-100, 0.5% sodium deoxycholate, 0.1% SDS) supplemented with 1 mM MgCl_2_, 1× HALT Protease Inhibitor Cocktail (Thermo Fisher) and 25 U/ml of Pierce Universal Nuclease for Cell Lysis (Thermo Fisher) and triturated through a 27G needle. After centrifugation (16 000 *g*, 15 min at 4°C), samples of the supernatant were collected, and insoluble pellets were washed with RIPA buffer and centrifuged as above. The samples were combined with 2× SDS sample buffer with 10% 2-mercaptoethanol and heated 5 min at 95°C. The samples were analysed by western blotting using the HSPB6 rb mAb [EPR14458]. HSPB6 level was expressed as the pellet-to-soluble ratio of HSPB6/total protein.

For filter trap assays (FTA), the cells were pelleted and lysed with FTA buffer (10 mM Tris–HCl, pH 8.0, 150 mM NaCl, 50 mM dithiothreitol) containing 2% SDS and 1× HALT, triturated through a 27G needle, and heated 5 min at 98°C. Soluble protein samples were removed from the lysates, combined with 2× SDS sample buffer containing 10% 2-mercaptoethanol, and heated 5 min at 95°C. To trap SDS-insoluble aggregates, samples of remaining lysates were filtered through a cellulose acetate membrane (pore size 0.2 μm, Whatman GmbH), followed by three washes with FTA buffer containing 0.1% SDS. Western blots of soluble protein samples and FTA membranes were stained with the HSPB6 rb mAb [EPR14458]. The ratio of SDS-insoluble (FTA) to soluble (WB) HSPB6 was calculated and normalized to that of wild-type HSPB6.

### HeLa immunofluorescence

HeLa cells seeded on coverslips were transfected with untagged wild-type or mutant HSPB6 constructs using FuGENE 6 (Promega). In coexpression experiments, V5-CRYAB or V5-HSPB1 were used in combination with an empty vector, or untagged wild-type or mutant HSPB6 at plasmid mass ratio 2:3. The cells were fixed two days post-transfection with 4% PFA/PBS or ice-cold methanol.

For analyses of microtubule content and stability, HeLa cells transfected as above were treated with 33 μM nocodazole for different times (2 min—1 h), placed on ice for 15 min, or left untreated. The cells were then pre-extracted for 1 min with 0.5% TX-100 in CBS (cytoskeletal buffer with sucrose—10 mM MES pH 6.1, 138 mM KCl, 3 mM MgCl_2_, 2 mM EGTA, 320 mM sucrose) at RT, and fixed for 15 min with 3.2% formaldehyde in CBS.

Indirect immunofluorescence stainings were done with antibodies against HSPB6, BAG3, β-tubulin, HSPB1, and the V5 tag. Actin filaments were stained with Alexa Fluor 488 Phalloidin (Thermo Fisher). Aggregated protein was visualized with the Proteostat Aggresome Detection Reagent (Enzo Life Sciences, Inc, Farmingdale, NY, USA). Widefield images were acquired with Zeiss Axio Imager M2 using 40× NA 1.3, 63× NA 1.25 and 100× NA 1.3 oil immersion objectives.

### Generation and culture of stable HSPB6-expressing HeLa cells

To create HeLa cells with cumate-inducible HSPB6 expression, HeLa cells were transfected with either a wild-type or p.Pro155Argfs^*^25;p.Gly163Arg HSPB6 PiggyBac construct in combination with the Super PiggyBac Transposase expression vector (System Biosciences PB210PA-1) using Lipofectamine 3000 (Thermo Fisher). Cells carrying the integrated constructs were selected with puromycin (1 μg/ml), and maintained as polyclonal pools for up to 8 passages in a medium containing 0.5 μg/ml puromycin. In the experiments, transgene expression was induced with 30 μg/ml of cumate, and medium containing a corresponding amount of ethanol was used as vehicle control. The induction and control media were changed daily during the experiments.

### RNA isolation and real-time polymerase chain reaction (RT-qPCR)

Stably transfected HeLa cells were collected after two days of cumate induction and stored in −80°C until use. RNA was extracted with Trizol (Invitrogen) according to the manufacturer’s instructions. cDNA synthesis was performed using SuperScript III Reverse Transcriptase (Invitrogen) and random primers, according to manufacturer’s protocol. RT-qPCR assays for *HSPB1* expression were performed using the iQ SYBR Green Supermix (Bio-Rad) and 25 nM of each specific primer. Each assay was performed with technical triplicates for each of the biological samples. 18S was used as reference gene for normalization. The primers used were F: 5′-CGTCGCTACTACCGATTGGATGG-3′ and R: 5′-TAATGATCCTTCCGCAGGTTCACCTAC-3′ for 18S, and F: 5′-GCGTGGTGGAGATCACCG-3′ and 5′-TTTACTTGGCGGCAGTCTCA-3′ for *HSPB1*. The *HSPB1* primers were confirmed not to amplify *HSPB1P1* or *HSPB1P2*.

### mRNA transfection of L6 myotubes

The mRNAs for wild-type and p.Pro155Argfs^*^25;p.Gly163Arg HSPB6 were transcribed *in vitro* from the respective SpeI-linearized pCR-Blunt II-TOPO constructs using the mMessage mMachine T7 Ultra Transcription Kit (Thermo Fisher).

Rat L6 myoblasts (ATCC CRL-1458) were plated on 35-mm dishes thin-coated with bovine collagen I (Thermo Fisher) and, after reaching confluency, differentiated to myotubes in ‘DMO’ (pyruvate-free DMEM with 2% heat-inactivated horse serum, 2 mM L-glutamine, penicillin/streptomycin, and 10% OPTI-MEM [Thermo Fisher]). The cells were transfected with HSPB6 mRNA (2.5 μg/dish) using the Lipofectamine MessengerMAX reagent (Thermo Fisher) after 3–4 days of differentiation, and on the following day fixed with 4% PFA/PBS. Indirect IF stainings were done with HSPB6 rb mAb [EPR14458] and CRYAB ms mAb G2JF, and widefield images acquired with Zeiss Axio Imager M2 using a 100× NA 1.3 oil immersion objective.

### Live cell microscopy

For live cell experiments, HeLa cells were grown on Lab-Tek II chambered coverslips and transfected with mTurquoise2-tagged and corresponding untagged HSPB6 constructs at a 1:3 ratio. Before imaging, the media was changed to FluoroBrite DMEM (Gibco) supplemented with 10% FCS and 1× GlutaMax. Time-lapse imaging was performed at 37°C, 5% CO_2_ using a Nikon Eclipse Ti-E widefield microscope and a 20× NA 0.75 objective. Images were acquired at 5-min intervals. For some of the long time-lapse experiments, 40 μg/ml cycloheximide was added to the medium to block synthesis of new proteins during the acquisition. In cell fusion experiments, cells were washed with PBS, treated with 50% (wt/vol) solution of PEG 1500 in PBS for 60 s, and washed several times with FluoroBrite DMEM +10% FCS before imaging. For studying dissolution of HSPB6 foci, the cells were treated with 5% (wt/vol) 1,6-hexanediol (1,6-HD) in FluoroBrite DMEM, and imaged ~ 30 seconds after 1,6-HD addition.

FRAP (fluorescence recovery after photobleaching) experiments were performed two days post-transfection on a Leica TCS SP8 confocal microscope and a 63× NA 1.20 water immersion objective at 37°C, 5% CO_2_. The mTurquoise2 fluorescence of a whole or partial HSPB6 droplet was bleached with high-intensity laser, after which fluorescence recovery was followed for 200 seconds at 5-s intervals.

### High-content analysis

For high-content analysis of HSPB1 expression, HeLa cells were grown on 96-well plates, transfected with untagged wild-type or variant pCDNA5/FRT/TO-HSPB6 constructs using FuGene 6, and fixed with 4% PFA after 2 d of expression. Indirect immunofluorescence staining was done with HSPB1 rb mAb EP1724Y (with secondary aRb-AF488) and HSPB6 ms mAb 6A4 (with secondary aMs-AF594). Nuclei and whole cells were visualized with Hoechst and CellMask Deep Red (Thermo Fisher). Images (3–6 fields per well) were acquired using the JOBS module on a Nikon Eclipse Ti-E widefield microscope and a 10× NA 0.30 objective. Image analysis was performed with CellProfiler 4.2.6. Briefly, cells were identified and cytoplasmic regions segmented based on nuclear and CellMask stainings. Illumination-corrected HSPB1 and HSPB6 images were background-corrected by subtracting the median intensity value of the area outside the cells from each image. Mean cytoplasmic HSPB1 and HSPB6 intensity values were quantified for each cell and normalized to the intensity value of the median cell in the image. Cells were assigned to high (>3× image median) or low (below image median) HSPB6, and low HSPB1 (<0.5× field image) intensity classes.

### Protein turnover assays

For assaying the turnover of variant HSPB6 proteins, HeLa cells were grown on 24-well plates and cotransfected with variant and wild-type HSPB6 constructs. For p.E139Rfs^*^41, p.P155Rfs^*^25;G163R and p.^*^161Wext^*^22, FuGene 6 transfection with 250 ng DNA/well (variant:wild-type ratio 50:50) was used. As the p.H110Pfs^*^108 protein could not be consistently detected with this setup, this variant was studied in separate experiments using Lipofectamine 3000 transfection with 500 ng DNA/well (variant:wild-type ratio 80:20). ~ 24 h after transfection, the cells were treated with cycloheximide (100 μg/ml) for up to 24 h to inhibit protein synthesis, and harvested to −80°C at the indicated time points. The levels of variant HSPB6 proteins were quantified from western blots and normalized to the wild-type protein serving as an internal control.

### Statistics

Statistical analyses were performed in GraphPad Prism 10.6.0. The Kruskal-Wallis test with Dunn’s multiple comparison test was used for comparing the group means.

## Supplementary Material

Sarparanta_SupplementaryFigs_150925_ddaf175

Sarparanta_SupplementaryTable_250425_ddaf175

## References

[1] Kampinga HH, de Boer H, Beerstra N. In: Tanguay R.M., Hightower L.E. (eds.), The Big Book on Small Heat Shock Proteins. Switzerland: Springer International Publishing, 2015, in press.

[2] Delbecq SP, Rosenbaum JC, Klevit RE. A mechanism of subunit recruitment in human small heat shock protein oligomers. Biochemistry 2015;54:4276–4284. 10.1021/acs.biochem.5b00490. 26098708 PMC4512712

[3] Kato K, Goto S, Inaguma Y. et al. Purification and characterization of a 20-kDa protein that is highly homologous to αB crystallin. J Biol Chem 1994;269:15302–15309. 10.1016/S0021-9258(17)36606-1. 8195168

[4] Verschuure P, Tatard C, Boelens WC. et al. Expression of small heat shock proteins HspB2, HspB8, Hsp20 and cvHsp in different tissues of the perinatal developing pig. Eur J Cell Biol 2003;82:523–530. 10.1078/0171-9335-00337. 14629120

[5] Golenhofen N, Perng MD, Quinlan RA. et al. Comparison of the small heat shock proteins αB-crystallin, MKBP, HSP25, HSP20, and cvHSP in heart and skeletal muscle. Histochem Cell Biol 2004;122:415–425. 10.1007/s00418-004-0711-z. 15480735

[6] van de Klundert FA, Smulders RH, Gijsen ML. et al. The mammalian small heat-shock protein Hsp20 forms dimers and is a poor chaperone. Eur J Biochem 1998;258:1014–1021. 10.1046/j.1432-1327.1998.2581014.x. 9990320

[7] Weeks SD, Baranova EV, Heirbaut M. et al. Molecular structure and dynamics of the dimeric human small heat shock protein HSPB6. J Struct Biol 2014;185:342–354. 10.1016/j.jsb.2013.12.009. 24382496

[8] Mymrikov EV, Riedl M, Peters C. et al. Regulation of small heat-shock proteins by hetero-oligomer formation. J Biol Chem 2020;295:158–169. 10.1074/jbc.RA119.011143. 31767683 PMC6952609

[9] Kato K, Shinohara H, Goto S. et al. Copurification of small heat shock protein with alpha B crystallin from human skeletal muscle. J Biol Chem 1992;267:7718–7725. 10.1016/S0021-9258(18)42574-4. 1560006

[10] Sugiyama Y, Suzuki A, Kishikawa M. et al. Muscle develops a specific form of small heat shock protein complex composed of MKBP/HSPB2 and HSPB3 during myogenic differentiation. J Biol Chem 2000;275:1095–1104. 10.1074/jbc.275.2.1095. 10625651

[11] Bukach OV, Seit-Nebi AS, Marston SB. et al. Some properties of human small heat shock protein Hsp20 (HspB6). Eur J Biochem 2004;271:291–302. 10.1046/j.1432-1033.2003.03928.x. 14717697

[12] Bukach OV, Glukhova AE, Seit-Nebi AS. et al. Heterooligomeric complexes formed by human small heat shock proteins HspB1 (Hsp27) and HspB6 (Hsp20). Biochim Biophys Acta 2009;1794:486–495. 10.1016/j.bbapap.2008.11.010. 19100870

[13] Mymrikov EV, Seit-Nebi AS, Gusev NB. Heterooligomeric complexes of human small heat shock proteins. Cell Stress Chaperones 2012;17:157–169. 10.1007/s12192-011-0296-0. 22002549 PMC3273557

[14] Wilhelmus MM, Boelens WC, Otte-Holler I. et al. Small heat shock proteins inhibit amyloid-beta protein aggregation and cerebrovascular amyloid-beta protein toxicity. Brain Res 2006;1089:67–78. 10.1016/j.brainres.2006.03.058. 16635482

[15] Bruinsma IB, Bruggink KA, Kinast K. et al. Inhibition of alpha-synuclein aggregation by small heat shock proteins. Proteins 2011;79:2956–2967. 10.1002/prot.23152. 21905118

[16] Mymrikov EV, Daake M, Richter B. et al. The chaperone activity and substrate Spectrum of human small heat shock proteins. J Biol Chem 2017;292:672–684. 10.1074/jbc.M116.760413. 27909051 PMC5241741

[17] Secco V, Tiago T, Staats R. et al. HSPB6: a lipid-dependent molecular chaperone inhibits alpha-synuclein aggregation. iScience 2024;27:110657. 10.1016/j.isci.2024.110657. 39280615 PMC11402235

[18] Fuchs M, Poirier DJ, Seguin SJ. et al. Identification of the key structural motifs involved in HspB8/HspB6-Bag3 interaction. Biochem J 2010;425:245–257. 10.1042/BJ20090907. 19845507

[19] Vos MJ, Zijlstra MP, Kanon B. et al. HSPB7 is the most potent polyQ aggregation suppressor within the HSPB family of molecular chaperones. Hum Mol Genet 2010;19:4677–4693. 10.1093/hmg/ddq398. 20843828

[20] Beall A, Bagwell D, Woodrum D. et al. The small heat shock-related protein, HSP20, is phosphorylated on serine 16 during cyclic nucleotide-dependent relaxation. J Biol Chem 1999;274:11344–11351. 10.1074/jbc.274.16.11344. 10196226

[21] Edwards HV, Scott JD, Baillie GS. PKA phosphorylation of the small heat-shock protein Hsp20 enhances its cardioprotective effects. Biochem Soc Trans 2012;40:210–214. 10.1042/BST20110673. 22260692 PMC3269834

[22] Komalavilas P, Penn RB, Flynn CR. et al. The small heat shock-related protein, HSP20, is a cAMP-dependent protein kinase substrate that is involved in airway smooth muscle relaxation. Am J Physiol Lung Cell Mol Physiol 2008;294:L69–L78. 10.1152/ajplung.00235.2007. 17993590 PMC2757925

[23] Vafiadaki E, Arvanitis DA, Eliopoulos AG. et al. The cardioprotective PKA-mediated Hsp20 phosphorylation modulates protein associations regulating cytoskeletal dynamics. Int J Mol Sci 2020;21:9572. 10.3390/ijms21249572. PMC7765622 33339131

[24] Chernik IS, Seit-Nebi AS, Marston SB. et al. Small heat shock protein Hsp20 (HspB6) as a partner of 14-3-3ɣ. Mol Cell Biochem 2007;295:9–17. 10.1007/s11010-006-9266-8. 17109079

[25] Dreiza CM, Brophy CM, Komalavilas P. et al. Transducible heat shock protein 20 (HSP20) phosphopeptide alters cytoskeletal dynamics. FASEB J 2005;19:261–263. 10.1096/fj.04-2911fje. 15598710

[26] Sluchanko NN, Artemova NV, Sudnitsyna MV. et al. Monomeric 14-3-3zeta has a chaperone-like activity and is stabilized by phosphorylated HspB6. Biochemistry 2012;51:6127–6138. 10.1021/bi300674e. 22794279 PMC3413243

[27] Sluchanko NN, Beelen S, Kulikova AA. et al. Structural basis for the interaction of a human small heat shock protein with the 14-3-3 universal Signaling regulator. Structure 2017;25:305–316. 10.1016/j.str.2016.12.005. 28089448 PMC5321513

[28] Fan GC, Chu G, Mitton B. et al. Small heat-shock protein Hsp20 phosphorylation inhibits beta-agonist-induced cardiac apoptosis. Circ Res 2004;94:1474–1482. 10.1161/01.RES.0000129179.66631.00. 15105294

[29] Qian J, Ren X, Wang X. et al. Blockade of Hsp20 phosphorylation exacerbates cardiac ischemia/reperfusion injury by suppressed autophagy and increased cell death. Circ Res 2009;105:1223–1231. 10.1161/CIRCRESAHA.109.200378. 19850943 PMC2799045

[30] Gardner GT, Travers JG, Qian J. et al. Phosphorylation of Hsp20 promotes fibrotic Remodeling and heart failure. JACC Basic Transl Sci 2019;4:188–199. 10.1016/j.jacbts.2018.11.007. 31061921 PMC6488766

[31] Karolczak-Bayatti M, Sweeney M, Cheng J. et al. Acetylation of heat shock protein 20 (Hsp20) regulates human myometrial activity. J Biol Chem 2011;286:34346–34355. 10.1074/jbc.M111.278549. 21803775 PMC3190820

[32] Chen A, Karolczak-Bayatti M, Sweeney M. et al. Lysine deacetylase inhibition promotes relaxation of arterial tone and C-terminal acetylation of HSPB6 (Hsp20) in vascular smooth muscle cells. Physiol Rep 2013;1:e00127. 10.1002/phy2.127. 24400135 PMC3871448

[33] Sudnitsyna MV, Sluchanko NN, Gusev NB. In: Tanguay R.M., Hightower L.E. (eds.), The Big Book on Small Heat Shock Proteins. Switzerland: Springer International Publishing, 2015, in press.

[34] Li F, Xiao H, Zhou F. et al. Study of HSPB6: insights into the properties of the multifunctional protective agent. Cell Physiol Biochem 2017;44:314–332. 10.1159/000484889. 29132139

[35] Tedesco B, Cristofani R, Ferrari V. et al. Insights on human small heat shock proteins and their alterations in diseases. Front Mol Biosci 2022;9:842149. 10.3389/fmolb.2022.842149. 35281256 PMC8913478

[36] Dreiza CM, Komalavilas P, Furnish EJ. et al. The small heat shock protein, HSPB6, in muscle function and disease. Cell Stress Chaperones 2010;15:1–11. 10.1007/s12192-009-0127-8. 19568960 PMC2866971

[37] Sudnitsyna MV, Seit-Nebi AS, Gusev NB. Cofilin weakly interacts with 14-3-3 and therefore can only indirectly participate in regulation of cell motility by small heat shock protein HspB6 (Hsp20). Arch Biochem Biophys 2012;521:62–70. 10.1016/j.abb.2012.03.010. 22450169

[38] Fan GC, Ren X, Qian J. et al. Novel cardioprotective role of a small heat-shock protein, Hsp20, against ischemia/reperfusion injury. Circulation 2005;111:1792–1799. 10.1161/01.CIR.0000160851.41872.C6. 15809372

[39] Fan GC, Zhou X, Wang X. et al. Heat shock protein 20 interacting with phosphorylated Akt reduces doxorubicin-triggered oxidative stress and cardiotoxicity. Circ Res 2008;103:1270–1279. 10.1161/CIRCRESAHA.108.182832. 18948619 PMC2763388

[40] Wang X, Zingarelli B, O'Connor M. et al. Overexpression of Hsp20 prevents endotoxin-induced myocardial dysfunction and apoptosis via inhibition of NF-kappaB activation. J Mol Cell Cardiol 2009;47:382–390. 10.1016/j.yjmcc.2009.05.016. 19501592 PMC2746739

[41] Liu GS, Zhu H, Cai WF. et al. Regulation of BECN1-mediated autophagy by HSPB6: insights from a human HSPB6(S10F) mutant. Autophagy 2018;14:80–97. 10.1080/15548627.2017.1392420. 29157081 PMC5846551

[42] Fan GC, Yuan Q, Song G. et al. Small heat-shock protein Hsp20 attenuates beta-agonist-mediated cardiac remodeling through apoptosis signal-regulating kinase 1. Circ Res 2006;99:1233–1242. 10.1161/01.RES.0000251074.19348.af. 17068291

[43] Wang X, Zhao T, Huang W. et al. Hsp20-engineered mesenchymal stem cells are resistant to oxidative stress via enhanced activation of Akt and increased secretion of growth factors. Stem Cells 2009;27:3021–3031. 10.1002/stem.230. 19816949 PMC2806498

[44] Stürner E, Behl C. The role of the multifunctional BAG3 protein in cellular protein quality control and in disease. Front Mol Neurosci 2017;10:177. 10.3389/fnmol.2017.00177. 28680391 PMC5478690

[45] Zamotina MA, Muranova LK, Zabolotskii AI. et al. Interaction of small heat shock proteins with BAG3. Biochimie 2025;232:15–24. 10.1016/j.biochi.2025.01.001. 39814164

[46] Fang X, Bogomolovas J, Wu T. et al. Loss-of-function mutations in co-chaperone BAG3 destabilize small HSPs and cause cardiomyopathy. J Clin Invest 2017;127:3189–3200. 10.1172/JCI94310. 28737513 PMC5531406

[47] Peng J, Li Y, Wang X. et al. An Hsp20-FBXO4 Axis regulates adipocyte function through modulating PPARgamma ubiquitination. Cell Rep 2018;23:3607–3620. 10.1016/j.celrep.2018.05.065. 29925002 PMC6091893

[48] Gao S, Zhang K, Zhou C. et al. HSPB6 deficiency promotes the development of aortic dissection and rupture. Lab Investig 2024;104:100326. 10.1016/j.labinv.2024.100326. 38237739

[49] Nicolaou P, Knöll R, Haghighi K. et al. Human mutation in the anti-apoptotic heat shock protein 20 abrogates its cardioprotective effects. J Biol Chem 2008;283:33465–33471. 10.1074/jbc.M802307200. 18790732 PMC2586274

[50] Shatov VM, Gusev NB. Physico-chemical properties of two point mutants of small heat shock protein HspB6 (Hsp20) with abrogated cardioprotection. Biochimie 2020;174:126–135. 10.1016/j.biochi.2020.04.021. 32353387

[51] Sarparanta J, Jonson PH, Kawan S. et al. Neuromuscular diseases due to chaperone mutations: a review and some new results. Int J Mol Sci 2020;21:1409. 10.3390/ijms21041409. PMC7073051 32093037

[52] Evilä A, Arumilli M, Udd B. et al. Targeted next-generation sequencing assay for detection of mutations in primary myopathies. Neuromuscular disorders : NMD 2016;26:7–15. 10.1016/j.nmd.2015.10.003. 26627873

[53] Sabaté R, Ventura S. In: Kister A.E. (ed.), Protein Supersecondary Structures. Totowa, NJ in press: Humana Press, 2013, 237–257.

[54] Peran I, Mittag T. Molecular structure in biomolecular condensates. Curr Opin Struct Biol 2020;60:17–26. 10.1016/j.sbi.2019.09.007. 31790873 PMC7117980

[55] Tedesco B, Vendredy L, Adriaenssens E. et al. HSPB8 frameshift mutant aggregates weaken chaperone-assisted selective autophagy in neuromyopathies. Autophagy 2023;19:2217–2239. 10.1080/15548627.2023.2179780. 36854646 PMC10351472

[56] Hardenberg M, Horvath A, Ambrus V. et al. Widespread occurrence of the droplet state of proteins in the human proteome. Proc Natl Acad Sci USA 2020;117:33254–33262. 10.1073/pnas.2007670117. 33318217 PMC7777240

[57] Vendruscolo M, Fuxreiter M. Sequence determinants of the aggregation of proteins within condensates generated by liquid-liquid phase separation. J Mol Biol 2022;434:167201. 10.1016/j.jmb.2021.167201. 34391803

[58] Heinrich S, Hondele M. Probing liquid-liquid phase separation of RNA-binding proteins In vitro and In vivo. Methods Mol Biol 2022;2537:307–333. 10.1007/978-1-0716-2521-7_18. 35895272

[59] Simon S, Fontaine JM, Martin JL. et al. Myopathy-associated αB-crystallin mutants: abnormal phosphorylation, intracellular location, and interactions with other small heat shock proteins. J Biol Chem 2007;282:34276–34287. 10.1074/jbc.M703267200. 17897943

[60] Nefedova VV, Datskevich PN, Sudnitsyna MV. et al. Physico-chemical properties of R140G and K141Q mutants of human small heat shock protein HspB1 associated with hereditary peripheral neuropathies. Biochimie 2013;95:1582–1592. 10.1016/j.biochi.2013.04.014. 23643870

[61] Nefedova VV, Sudnitsyna MV, Strelkov SV. et al. Structure and properties of G84R and L99M mutants of human small heat shock protein HspB1 correlating with motor neuropathy. Arch Biochem Biophys 2013;538:16–24. 10.1016/j.abb.2013.07.028. 23948568

[62] Fujita Y, Ohto E, Katayama E. et al. alphaB-crystallin-coated MAP microtubule resists nocodazole and calcium-induced disassembly. J Cell Sci 2004;117:1719–1726. 10.1242/jcs.01021. 15075233

[63] Houck SA, Clark JI. Dynamic subunit exchange and the regulation of microtubule assembly by the stress response protein human alphaB crystallin. PLoS One 2010;5:e11795. 10.1371/journal.pone.0011795. 20668689 PMC2909917

[64] Almeida-Souza L, Asselbergh B, De Winter V. et al. HSPB1 facilitates the formation of non-centrosomal microtubules. PLoS One 2013;8:e66541. 10.1371/journal.pone.0066541. 23826100 PMC3691211

[65] Ohto-Fujita E, Hayasaki S, Atomi A. et al. Dynamic localization of alphaB-crystallin at the microtubule cytoskeleton network in beating heart cells. J Biochem 2020;168:125–137. 10.1093/jb/mvaa025. 32725133

[66] Belanto JJ, Olthoff JT, Mader TL. et al. Independent variability of microtubule perturbations associated with dystrophinopathy. Hum Mol Genet 2016;25:4951–4961. 10.1093/hmg/ddw318. 28171583 PMC6078591

[67] Randazzo D, Khalique U, Belanto JJ. et al. Persistent upregulation of the beta-tubulin tubb6, linked to muscle regeneration, is a source of microtubule disorganization in dystrophic muscle. Hum Mol Genet 2019;28:1117–1135. 10.1093/hmg/ddy418. 30535187 PMC6423419

[68] Lucas L, Cooper TA. Insights into cell-specific functions of microtubules in skeletal muscle development and homeostasis. Int J Mol Sci 2023;24:2903. 10.3390/ijms24032903. PMC9917663 36769228

[69] Almeida-Souza L, Asselbergh B, d'Ydewalle C. et al. Small heat-shock protein HSPB1 mutants stabilize microtubules in Charcot-Marie-tooth neuropathy. J Neurosci 2011;31:15320–15328. 10.1523/JNEUROSCI.3266-11.2011. 22031878 PMC6703512

[70] Zhang X, Qiao Y, Han R. et al. A Charcot-Marie-tooth-causing mutation in HSPB1 decreases cell adaptation to repeated stress by disrupting autophagic clearance of misfolded proteins. Cells 2022;11:2886. 10.3390/cells11182886. PMC9496658 36139461

[71] Vicart P, Caron A, Guicheney P. et al. A missense mutation in the alphaB-crystallin chaperone gene causes a desmin-related myopathy. Nat Genet 1998;20:92–95. 10.1038/1765. 9731540

[72] Graw J . Genetics of crystallins: cataract and beyond. Exp Eye Res 2009;88:173–189. 10.1016/j.exer.2008.10.011. 19007775

[73] Cortese A, Curro R, Ronco R. et al. Mutations in alpha-B-crystallin cause autosomal dominant axonal Charcot-Marie-tooth disease with congenital cataracts. Eur J Neurol 2024;31:e16063. 10.1111/ene.16063. 37772343 PMC10872581

[74] Gorter RP, Stephenson J, Nutma E. et al. Rapidly progressive amyotrophic lateral sclerosis is associated with microglial reactivity and small heat shock protein expression in reactive astrocytes. Neuropathol Appl Neurobiol 2019;45:459–475. 10.1111/nan.12525. 30346063 PMC7379307

[75] Dubowitz V, Sewry CA. Muscle Biopsy: A Practical Approach. Philadelphia, USA: Saunders Elsevier, 2007.

[76] Ávila-Polo R, Malfatti E, Lornage X. et al. Loss of Sarcomeric scaffolding as a common baseline histopathologic lesion in titin-related myopathies. J Neuropathol Exp Neurol 2018;77:1101–1114. 10.1093/jnen/nly095. 30365001

[77] Schindelin J, Arganda-Carreras I, Frise E. et al. Fiji: an open-source platform for biological-image analysis. Nat Methods 2012;9:676–682. 10.1038/nmeth.2019. 22743772 PMC3855844

