# Supplementary material for: C-terminal extension of HSPB6 in a family with myopathy and cataract
Source: Hum Mol Genet. 2025 Nov 26;35(1):ddaf175. doi: 10.1093/hmg/ddaf175 (PMC13158247; doi:10.1093/hmg/ddaf175)
Supplement: Sarparanta_SupplementaryFigs_150925_ddaf175 [file sarparanta_supplementaryfigs_150925_ddaf175.pdf]

Sarparanta et al. **C-terminal extension of HSPB6 in a family with myopathy and cataract**

Supplementary Figures

Supplementary Figure 1

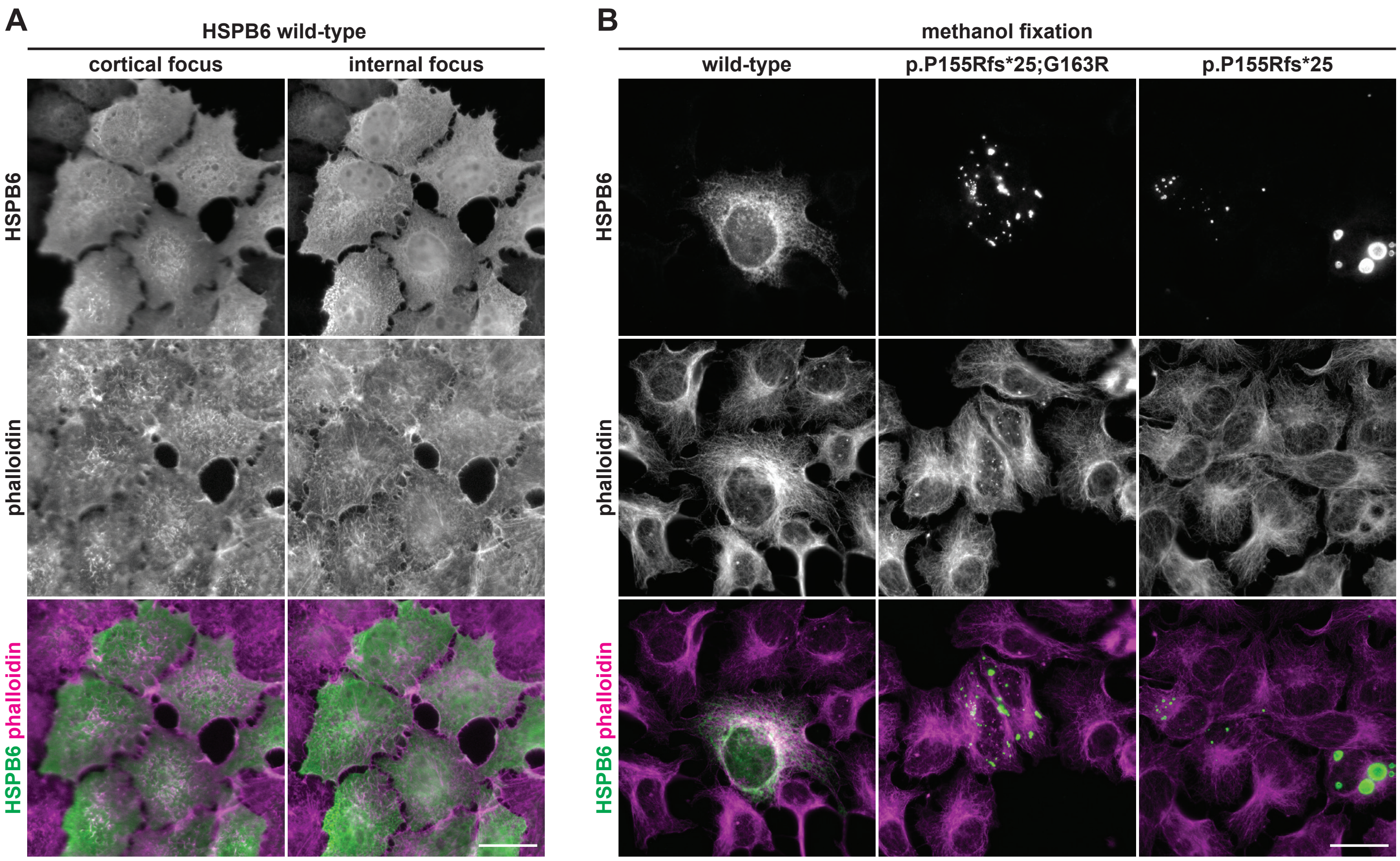

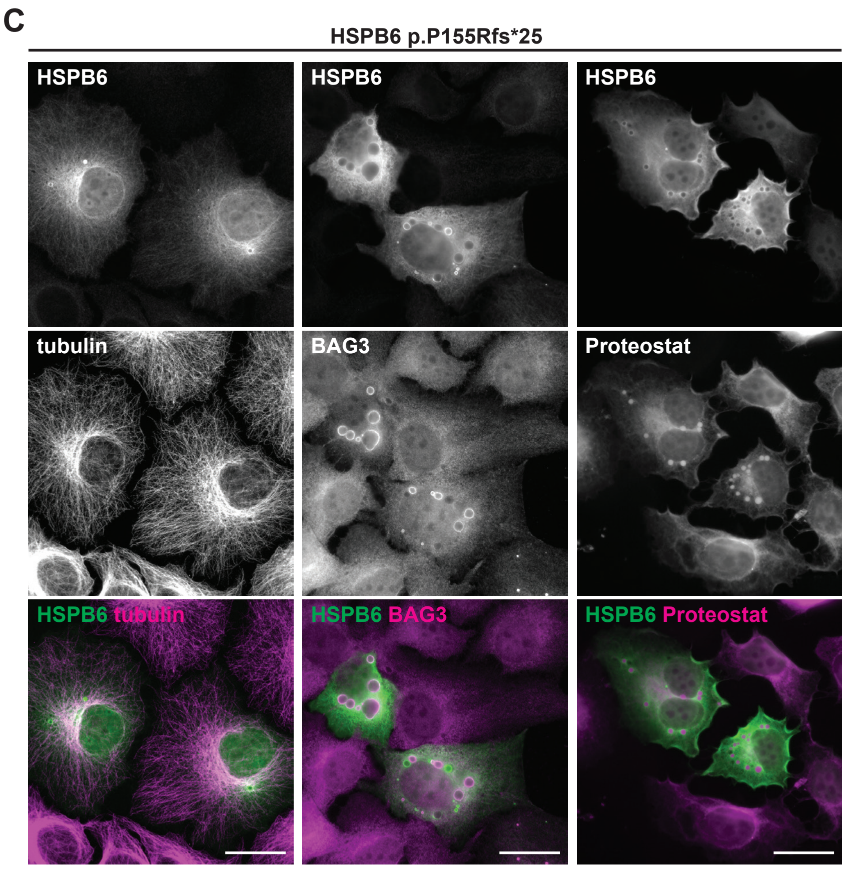

A) Association of wild-type HSPB6 with cortical actin cytoskeleton. HeLa cells transfected with the wild-type HSPB6 construct were stained for HSPB6 and actin filaments (phalloidin). Image focused at the cortical level shows colocalization of HSPB6 staining and the actin meshwork.

B) HeLa cells expressing wild-type or mutant HSPB6, fixed with methanol and stained for HSPB6 and tubulin.

C) HeLa cells expressing HSPB6 p.P155Rfs*25, stained for HSPB6, tubulin, BAG3, and the Proteostat dye as indicated. Compare to wild-type and p.P155Rfs*25;G163R in Fig. 5.

Scale bars 20 µm.

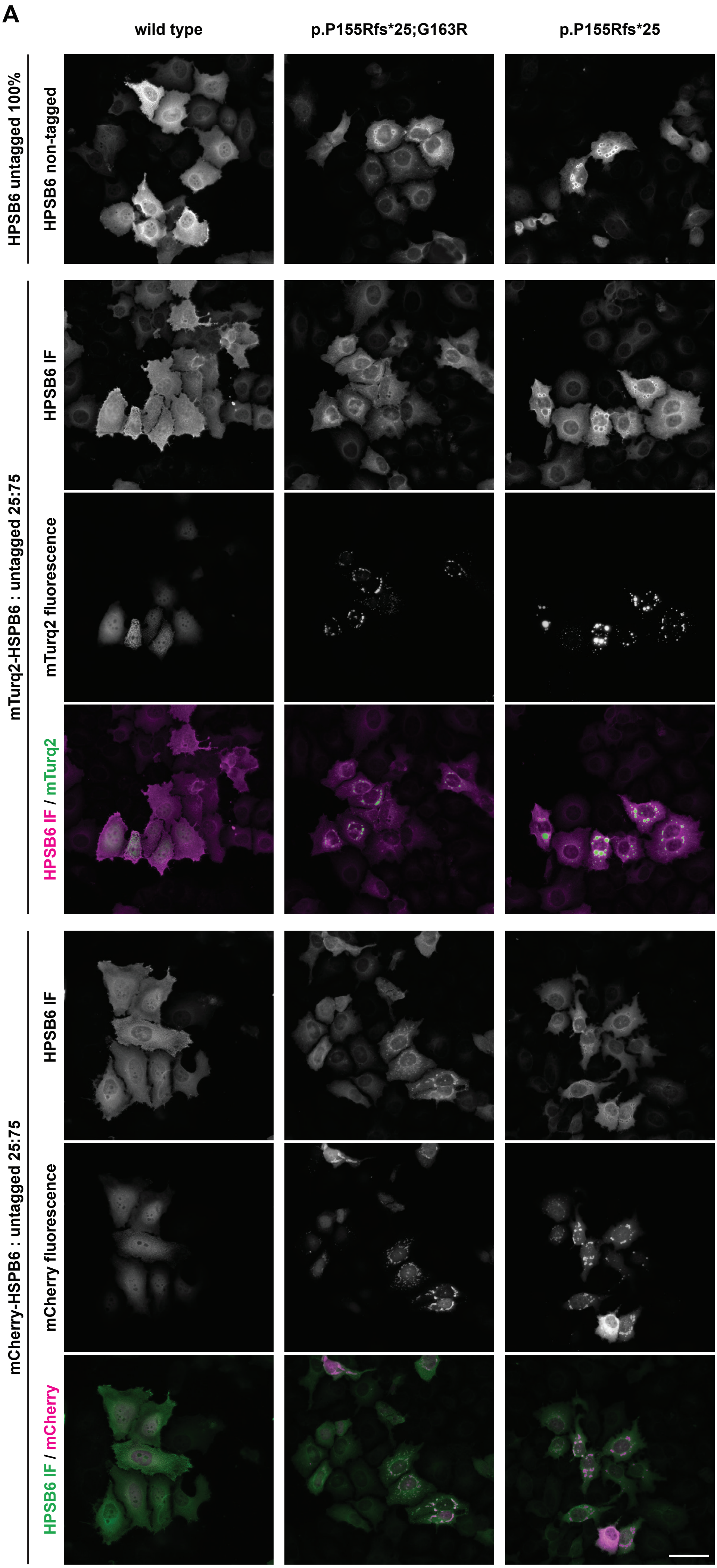

Supplementary Figure 2

A) Validation of fluorescent HSPB6 constructs. HeLa cells cotransfected with mTurquoise2 (mTurq2) - or mCherry- tagged HSPB6 constructs and the corresponding untagged constructs were PFA-fixed and immunostained for HSPB6. Comparison of mTurq2 and mCherry fluorescence to HSPB6 IF staining allows evaluating the effects of the tags on protein behavior. The mTurq2-tagged constructs showed exaggerated localization to mutant HSPB6 condensates but did not alter their appearance compared to cells transfected with untagged HSPB6 alone. The mCherry tag altered condensate morphology and was excluded from further studies. Scale bar 50 µm.

B) HeLa cells expressing wild-type mTurq2-HSPB6 were imaged live and after PFA fixation. The granular pattern appeared during fixation.

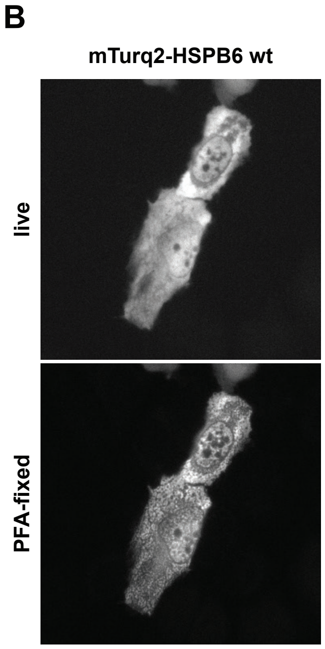

Supplementary Figure 3

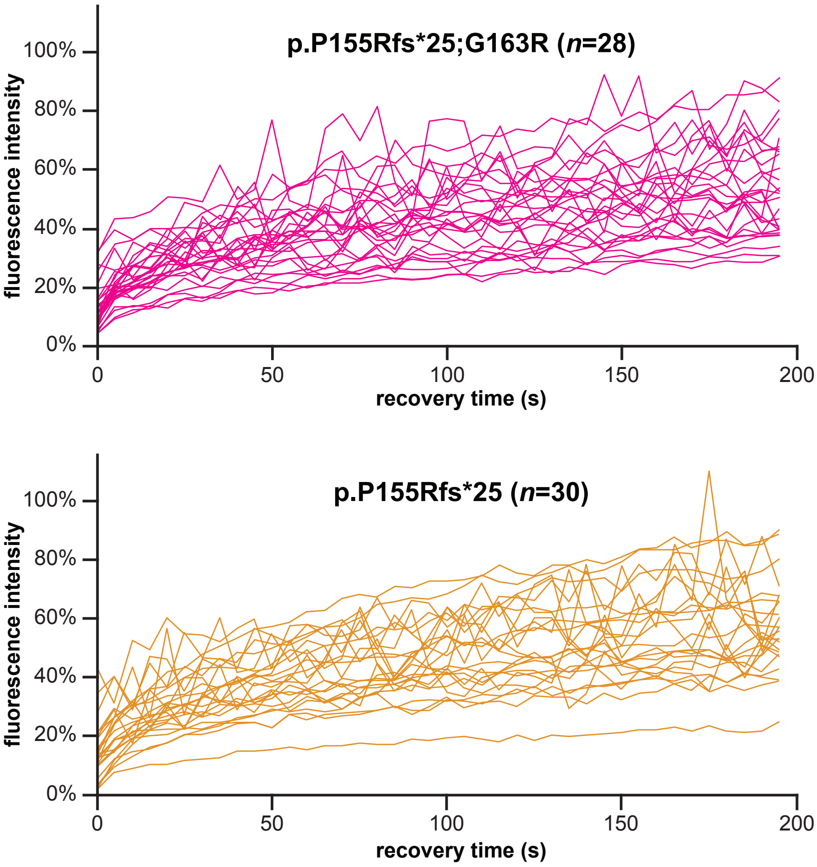

FRAP curves for HSPB6 p.P155Rfs*25;G163R and p.P155Rfs*25 spherical foci demonstrate variable fluorescence recovery of the foci. HeLa cells were transiently transfected with mTurquoise2-tagged and untagged HSPB6 constructs at a 1:3 ratio and analyzed two days post-transfection. The graphs show data for *n*=28–30 foci from four independent experiments.

Supplementary Figure 4

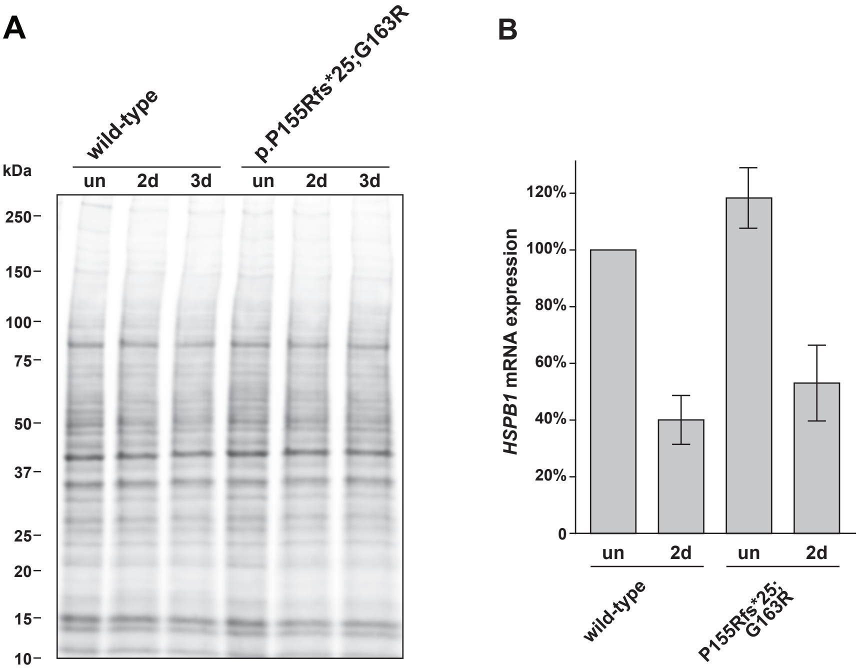

A) Total protein staining for the western blot presented in Fig. 7D.

B) Stably transfected HeLa cells were induced to express the HSPB6 constructs for 2 days or left uninduced (un), and *HSPB1* mRNA expression was analyzed by RT-qPCR. The graph shows mean ± SD of the mean expression from three experiments, performed in parallel to the protein analyses presented in Fig. 7D–E.

Supplementary Figure 5

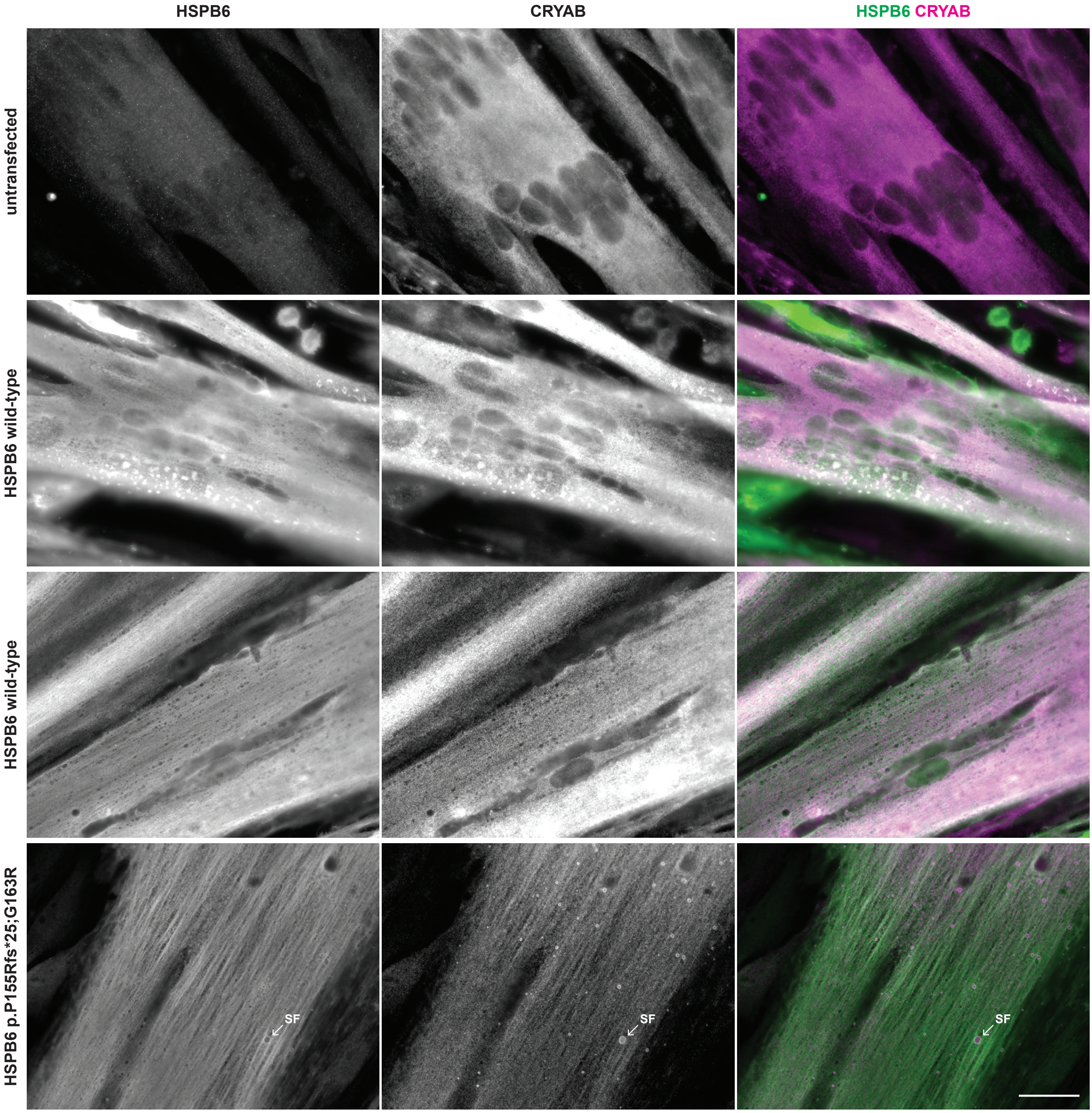

L6 myotubes transfected with *HSPB6* mRNA (wild-type or p.P155Rfs*25;G163R), stained for HSPB6 and CRYAB. Rare myotubes expressing the variant construct showed spherical foci (SF) that recruited CRYAB. Scale bar 20 µm.

Supplementary Figure 6

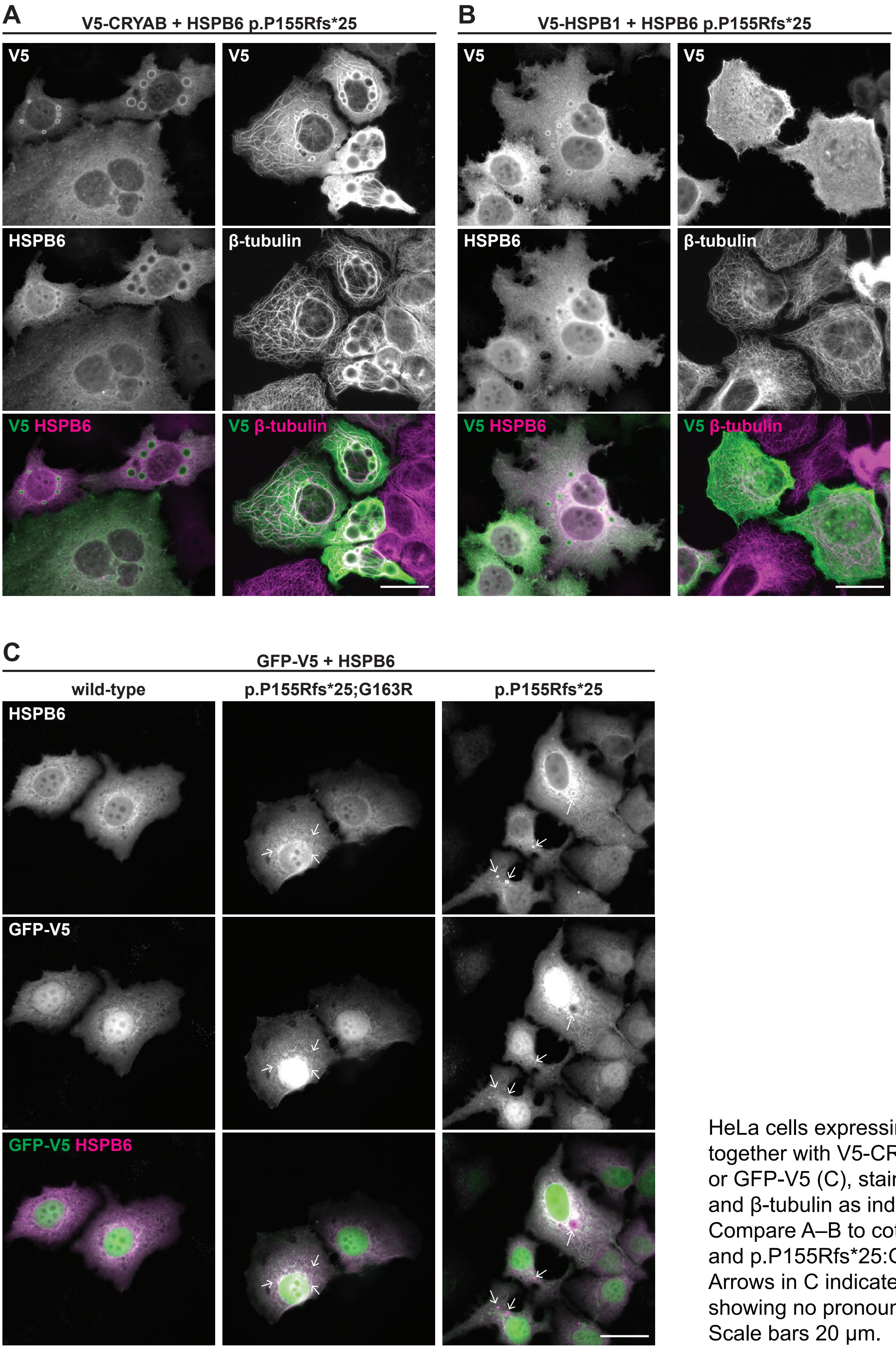

HeLa cells expressing HSPB6 constructs together with V5-CRYAB (A), V5-HSPB1 (B) or GFP-V5 (C), stained for the V5 tag, HSPB6 and β-tubulin as indicated.
Compare A–B to cotransfections with wild-type and p.P155Rfs*25:G163R HSPB6 in Fig. 8.
Arrows in C indicate spherical HSPB6 foci, showing no pronounced GFP-V5 recruitment.
Scale bars 20 µm.

Supplementary Figure 7

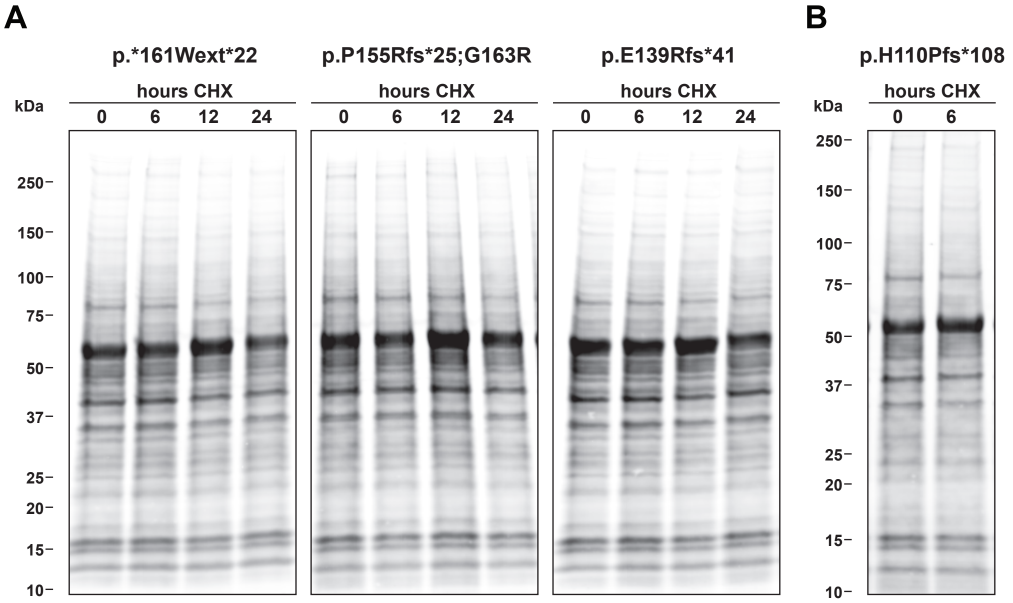

Total protein stainings for the western blots presented in Fig. 10B **(A)** and Fig. 10D **(B)**.
